# Regional trade of medicinal plants has facilitated the retention of traditional knowledge: case study in Gilgit-Baltistan Pakistan

**DOI:** 10.1186/s13002-018-0281-0

**Published:** 2019-01-28

**Authors:** Muhammad Asad Salim, Sailesh Ranjitkar, Robbie Hart, Tika Khan, Sajid Ali, Chandni Kiran, Asma Parveen, Zahra Batool, Shanila Bano, Jianchu Xu

**Affiliations:** 1 Key Laboratory for Plant Diversity and Biogeography of East Asia, Kunming Institute of Botany, Chinese Academy of Sciences, Kunming, 650201 Yunnan China; 2 University of Chinese Academy of Sciences, Beijing, 100049 China; 3 World Agroforestry Centre (ICRAF), East and Central Asia Office, Kunming, 650201 Yunnan China; 4 Missouri Botanical Garden, Post Office Box 299, St. Louis, MO 63166 USA; 5 Department of Biological Sciences, Karakorum International University, Gilgit, Gilgit-Baltistan Pakistan

**Keywords:** Ethnomedicine, Ethnoecology, Medicinal plants, Traditional knowledge, Trade in medicinal plants, Gilgit-Baltistan

## Abstract

**Background:**

The ethnic groups in Gilgit-Baltistan have been utilizing local resources in their centuries-old traditional healing system. Most tribes within these ethnic groups still rely on traditional healing systems. We aim to understand the current status, uses, and abundance of medicinal plants, associated traditional knowledge, and trade.

**Materials and methods:**

The study incorporated over 300 local community members (70% men and 30% women) in focused group discussions, semi-structured interviews, and homework assignments for 8th to 12th grade students to document traditional knowledge (TK) in six districts in Northeast Pakistan. We calculated various indices such as informant consensus factor, use value, relative frequency of citation, and CoKriging. These indices, along with repetitively used medicinal plants, were used to analyze differences in studied locations.

**Results:**

Most of the community members still rely on traditional medication in the study areas. However, we found the highest number of medicinal plants used in Skardu and Gilgit compared to other districts and these two districts also represent trade centers and a highly populated area regarding medicinal plants. Results indicate connection amongst the surveyed villages signifying mixing of knowledge from different sources, with certain areas more influenced by traditional Chinese medicine and others more by Ayurveda and Unani.

**Conclusion:**

TK is mostly retained with elder community members; however, those directly linked with market value chain retain rich knowledge on traditional use of the medicinal plants from the region. Major trade centers in the region also coincide with a high density of medicinal plant occurrence, knowledge, and higher utilization. Therefore, with the increasing trade in medicinal plant in the region, there is potential for rejuvenation of this knowledge and of plant use in the region.

**Electronic supplementary material:**

The online version of this article (10.1186/s13002-018-0281-0) contains supplementary material, which is available to authorized users.

## Introduction

Gilgit-Baltistan, the northeast mountainous region of Pakistan, is outstanding for its rich ethnic diversity [1–3]. This region is situated in a strategic geographical location that is important as a part of the ancient silk route and its position along the China-Pakistan Economic Corridor [3–5]. For centuries, knowledge exchange has occurred between indigenous dwellers and migrants and peddlers from the southern, northern, and western parts of Asia. Besides, the region is one of the important plant areas in the Himalaya, Karakoram, and Hindukush (HKH) landscape [6–12]. In the epoch of Anthropocene, as for other parts of the world, this region also experienced problems due to human population growth and associated land use transformation that severely affected both important species and the wider ecosystem [13–15]. Still natural resources, especially plant diversity, are very significant for ethnic communities in this mountainous landscape [10, 14, 16, 17]. Plants are the source of energy and food, a building material for houses, and a main component of the health care system as folklore medicine [18–27].

There are about 600 species of flowering plants in Pakistan that are utilized as medicinal plants and around 500 of these have global significance and studies available [9, 16, 28]. Around 50,000 traditional healers and informal Pansaris (retailers) are registered in Pakistan who frequently utilize and sell 400–600 plants species for their medicinal, cultural, traditional, and spiritual benefits [29–31]. Gilgit-Baltistan, with above 300 reported species of MAPs, is a hotspot for medicinal plants and their utilization in Pakistan [9, 12–14, 16, 27, 30, 32–41]. With seven districts and an ideal mountainous landscape, this region is naturally suitable for high-value medicinal plants. The local communities have been utilizing this resource for many generations [9, 12, 15, 33, 36, 42–45]. Several researchers have previously documented traditionally used medicinal plant from different districts in this region. Shedayi and Gulshan (2012) and Shedayi et al. (2014) in Ghizer district; Khan et al. (2013) and Akhtar et al. (2016) in Hunza; Bano et al. (2014) in Skardu; Khan and Khatoon (2007) and Fahad and Bano (2012) in Gilgit; Abbas et al. (2016) in Baltistan and the contributions of Hussain et al. (2011), Khan et al. (2011), Qureshi et al. (2006), and Ali et al. (2017) are important and noteworthy for the documentation of plant species used locally for medicinal purposes besides reporting on the modes of their uses and diseases targeted/cured through traditional herbal practitioners.

In addition to the utilization of medicinal plants in the traditional healing system, trade in herbal raw material and product is not new in the area. As this region serves as an ancient trade route that connects south Asia with China, Central Asia, and West Asia; trade in medicinal plants and exchange of traditional healing knowledge is very likely. For instance, archeological studies reveal the influence of cultural incursions from the Indian subcontinent, China, Scythia (Eurasia), Transoxiana (Uzbekistan, Tajikistan, southern Kyrgyzstan, and southwest Kazakhstan), and Ancient Greece, amongst others on traditional medicinal system [3, 46]. Before the introduction of Islam, the communities in Gilgit-Baltistan were predominantly practicing Buddhism [3, 46–50]. The region is recognized for its contributions towards survival and expansion of Ayurveda during the British regime [51]. Hakim Ajmal Khan is a famous Indian physician who worked for the revival of Ayurveda and Unani systems during the British era by establishing an Ayurveda and Unani medical college and a pharmaceutical company besides continuing with his own clinical practice of the systems [52]. Before the region completely came under Dogra Raj of the Kashmir State, Hunza, Gilgit, Nagar, and Ghizer mainly remained under Chinese influence, whereas Skardu, Astore, Ghanche, and Diamer remained under Tibetan influence [3, 46]. Recently, with development of the Karakoram Highway (KKH) and China-Pakistan Economic Corridor (CPEC) that follow the ancient Silk Routes [3, 50] in the region, this area has become important junction of trade.

Our research provides the first comparative study for six districts of Gilgit-Baltistan where the influence of Chinese, Ayurveda, Unani, and Tibetan healing systems on folk knowledge has been observed. Traditional knowledge of medicinal plants is often socially integrated through communal learning and intercultural exchange. Medicinal plants in mountainous terrain are known for their distribution in elevation corridors or endemism to a particular locality. We therefore aim to explore the richness of traditional knowledge of medicinal plants, their uses, distribution, and trade in the mountainous region of Gilgit-Baltistan, learn about the knowledge exchange between the old and young generation as well as amongst different communities and localities of the region, and how medicinal systems like Chinese, Ayurveda, Tibetan, and Unani influenced the use of traditional medicine system in the region. We also explore the possible factors behind the general decline in knowledge about medicinal plants, yet the continued use of traditional medicine for treatment of different diseases and how the current markets and market players supplement this phenomenon. We take an inventory of these plants, their use value, and local importance at a regional level and current markets. We also look at how these factors and the influence of different medicinal systems in the region compliment the transfer of knowledge to younger generations and across different ethnicities and locations in the region.

## Materials and methods

### Study area

Gilgit-Baltistan is located in the far north of Pakistan, with Afghanistan to the north and west, China to the north and east, and India to the south [8]. The seven districts are spread over an area of 72,496 km^2^. The region is rugged and mountainous, located amongst three of the highest mountain ranges—the Himalayas, the Karakoram, and the Hindukush (HKH) and home to the largest number of glaciers outside the polar region [36, 53, 54].

**Fig. 1:**
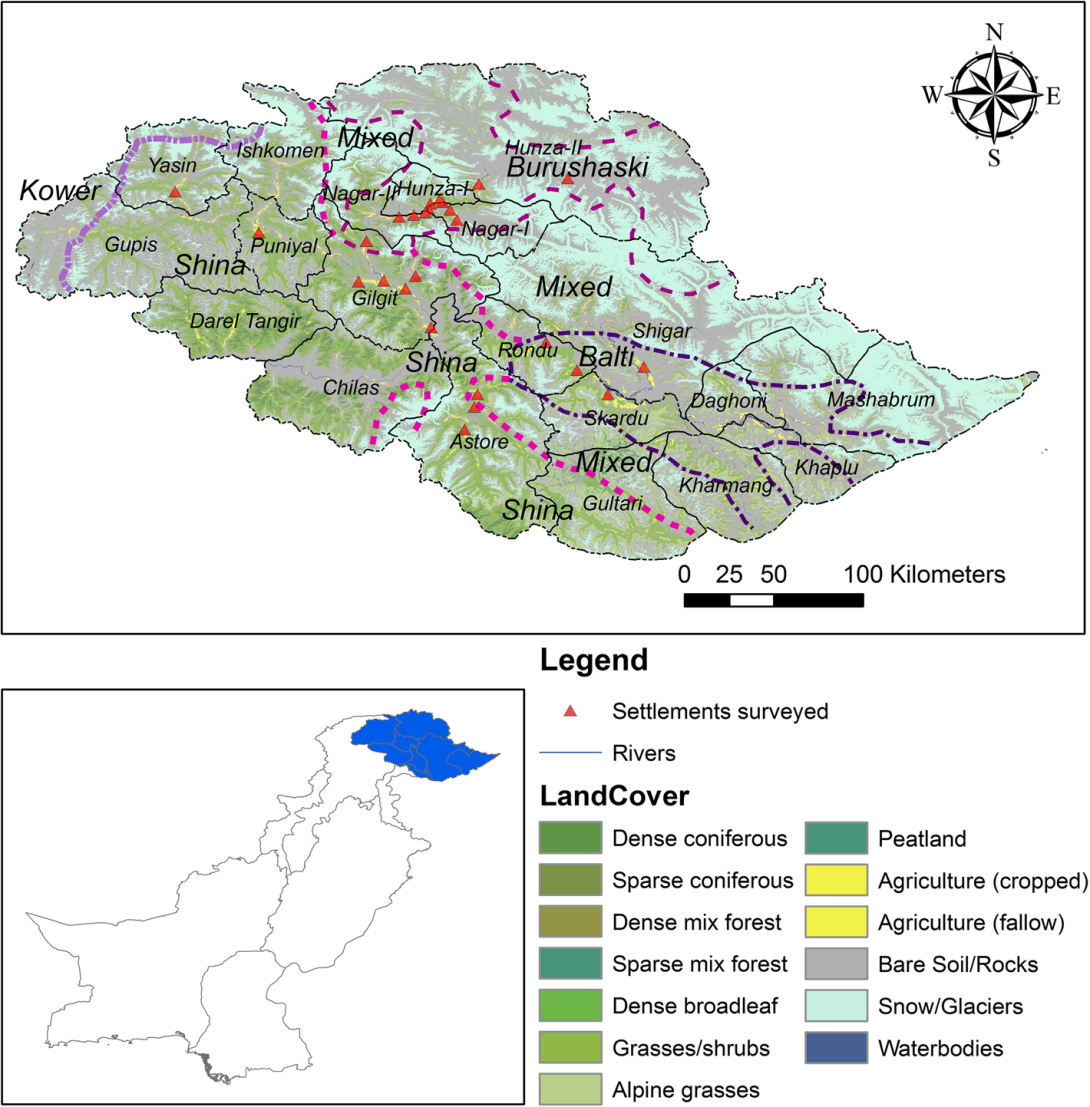
Area map showing vegetative cover, settlements, center of major ethno-linguistic clusters, and surveyed areas in Gilgit-Baltistan

The study was focused on Gilgit, Hunza, Nagar, Ghizer, Skardu, and Astore Districts of Gilgit-Baltistan. The residents are divided into sub-groups based on their origin as well as their ethno-linguistic clustering (Fig. 1). Yashkun, Sheen/Shinaki, Wakhi (those who migrated from Wakhan), Burushos, Dom, Brokpa, and Balti are the main tribes of the area; some Kashmiris, Kohistani, Mongols, Mughals, Rajas, Pathans, Gujar, Soniwal, Mon, Hor, and Kashgari also reside here [35, 55–60]. The majority of Gilgit-Baltistan is sparsely populated with these tribes, but Ghizer is dominated by Burushos; Gilgit, Hunza, and Nagar have Burushos, Sheen, and Yashkun; Diamer and Astore are majorly populated by Sheen, Yashkun, and Kohistani communities; and Skardu and Ghanche are predominantly Mon, Hor, and Mongols [60, 61]. The languages spoken by Burushos, Sheen, and Yashkun are Shina, Burushaski, Wakhi, and Khowar (only Ghizer and parts of Hunza) while the Mongols, Mon, and Hor speak Balti [9, 38, 60, 62].

Communities in Gilgit-Baltistan are dependent on agricultural resources and live close to the forest area (Fig. 1). Land cover changes, lack of resource management and sustainable harvesting policies, and political interests at massive scale in the HKH and Pamir mountain ranges have severe and long-lasting impacts on the region [63–66]. Medicinal plants and traditional medication have been used for generations for curing different diseases [34, 36, 63, 67]. Since opening of KKH in 1982 [4], markets for local products started to develop, thus exerting pressure on natural resources [33, 68].

### Ethnobotanical survey

The study was carried out from January 2017 to May 2018, during which 300+ local community members were approached via focused group discussions (FGDs), semi-structured interviews, and homework assignments for 8th to 12th Grade school and college students. The participants included men and women community members, local traditional health practitioners (THPs), community elders, and wholesalers and retailers of medicinal plants. Following the snowball sampling methodology [69–74], a total of 15 FGDs and 240 individual interviews were conducted for data collection. Sixty students were provided with a set of questionnaires to reach their families and understand what kind of plants/herbal remedies are used in the families and how traditional knowledge is preserved within a family. This method was tested in Nepal and was quite effective to document traditional knowledge [75]. Prior permission and consent for data collection and publication was obtained from all the participants. The homework assignments for school students were administered under the supervision of concerned class teachers. Data on age and gender was also acquired from the participants. These methods resulted in data covering an ethnobotanical inventory of plants, part used, therapeutic utilities, the location and timing of acquiring the resource, and the existing markets with trade opportunities. The initial taxonomic identification of medicinal plants was done by a taxonomist in the field [76–78] and by cross-referencing photographs, voucher specimens, and the local name of species with previously available material and literature from the study area [9, 35, 41, 79, 80]. Information on local names of plant species, and parts of the plant utilized for different medicinal purposes was recorded. Collected specimens were mounted on herbarium sheets and identified by the taxonomists in the Karakoram International University. The voucher specimens were authenticated through the plant list (www.theplantlist.org), tropicos (www.tropicos.org), and flora of Pakistan (www.efloras.org) and deposited in the herbarium of the Karakoram International University.

### Quantitative analysis

We regressed age of participants against the number of species reported by respective age group and people engaged in the trade of medicinal plants during the survey to understand TK in the region. We used CoKriging method [81] to interpolate the number of species recorded and used in various medical conditions from the surveyed locations to recognize section of highest medicinal plant use in the region. Kriging is an advanced geostatistical procedure that generates an estimated surface from a scattered set of points with *z* values. CoKriging is multivariate extension of Kriging method that uses information from one or more correlated variables measured in the same range. This method is useful in mountainous areas with rugged terrain. This method has been tested and used in tree richness mapping, abundance mapping, and recognizing areas at high risk of species invasion [81–83].

We calculated quantitative measures like informant consensus factor (ICF), use value (UV), and relative frequency of citation (RFC) for medicinal plants based on 208 illnesses categorized into 29 ailment groups from each district in the survey. The illnesses were categorized based on a particular part of the body affected or particular illnesses for multiple parts of the body. For example, all kinds of skin illnesses were categorized under skin infections while diseases related to stomach and intestinal disorders were grouped into stomach and intestine category (further detail is available in Additional files 1, 2, 3, 4, 5, 6, 7, 8, 9, 10, 11, and 12). A brief description of the quantitative measures is provided below:

#### Informant consensus factor (ICF)

The ICF was calculated to find a consensus between participants on the reported treatments for each set of diseases [79, 84, 85]. ICF was calculated using given formula [79, 86, 87];

$$ \mathrm{ICF}=\frac{\left(\mathrm{Nur}-\mathrm{Nt}\right)}{\left(\mathrm{Nur}-1\right)} $$

where Nur indicates the number of use reports for a specific disease category and Nt mentions the number of taxa used for the disease category.

#### Use value (UV)

Use value determines the quantitative measure for relatively important local plant species [78, 87–89]. The use value is calculated using the following formula:

$$ \mathrm{UV}=\frac{\sum \mathrm{Ui}}{N} $$

where∑Ui is the total number of use reports for a given species and *N* is the total number of participants inquired for the species.

#### Relative frequency of citation (RCF)

Ethno medicinal data was quantitatively analyzed using RFC which indicated the local importance of medicinal species. The RFC was calculated using given formula [87, 89, 90]:

$$ \mathrm{RFC}=\frac{\mathrm{FC}}{N}\left(0<\mathrm{RFC}<1\right) $$

where FC is the number of participants reporting on the use of a plant species and *N* is the total number of participants in the survey.

#### Discriminant analysis (DA)

We used discriminant analysis to delimit the geographical differences of the ethnobotanical knowledge using quantitative measures (viz. UV and RCF), highly used species, and number of treatments. Discriminant function analysis or discriminate analysis (DA) determines naturally occurring groups and the variables responsible for segregating amongst them [91–93].

## Results and discussion

### Demographic features of the participants

**Fig. 2:**
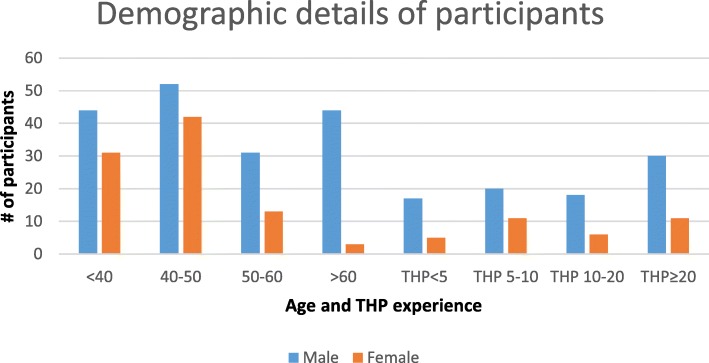
**a** Comparison of medicinal plant species highest occurrence and uses in Gilgit-Baltistan region. **b** Linear regression showing relation of age and number of plant species reported. Legend TK = participants and Trade = retailers and traditional healers

**Fig. 3:**
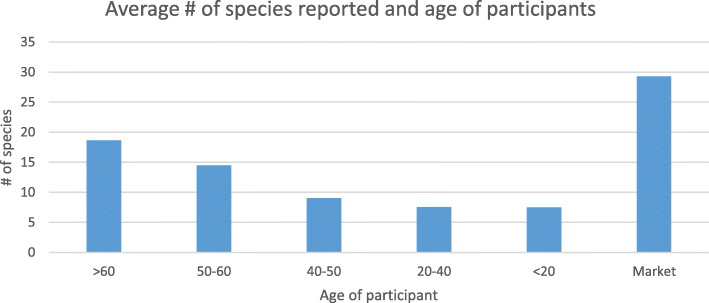
Section of the highest use and occurrence of medicinal plants and uses in Gilgit-Baltistan region

**Fig. 4:**
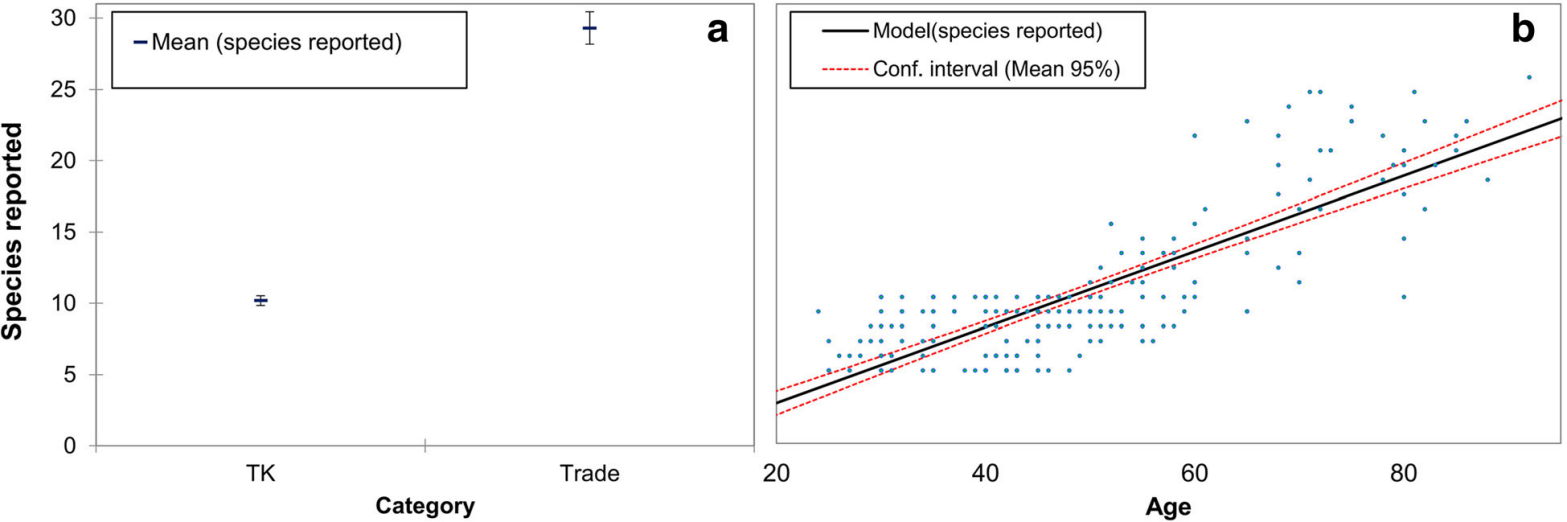
Grouping survey sites in Gilgit-Baltistan based on the medicinal plants and their use reports

More than 300 participants including 70% men and 30% women were interviewed during the survey (Fig. 2). The Yashkun, Sheen/Shinaki, Burushos, Wakhi, and Balti communities approached during the survey possessed good knowledge on medicinal plants use. Although these tribes maintain individual identities representing different parts of the mountainous region, the cross-cultural interactions have led to the growth and diversification of the traditional knowledge. The results from FGDs, HH, and market surveys revealed that regardless of ethnicity, THPs and retailers retain a significant level of information on the medicinal plants of the region, the locations from where the plants can be acquired and the ailments they can be utilized for. Although the participants from the community and THPs provided more extensive information than the retailers, when it came to the question of how to use a certain plant as medicine, the retailers had ample information to share on a higher number of medicinal plants and their general uses (Fig. 3 Fig. 4a). We found that participants above 50 years of age had significant traditional knowledge regarding utilization of medicinal plants. This was evident from the number of species and their uses reported per interview where a higher number of species was reported by participants above 50 years of age. Our results from the linear regression (*R*^2^ = 0.65; *p* < 0.0001) also revealed that number of species reported increase with age of the participant (Fig. 4b). The students responding to homework assignments mostly brought information from women representatives of the household, thus providing relevant information on which plant species are kept at home and used for treating common sicknesses.

### Taxonomic diversity

**Fig. 5:**
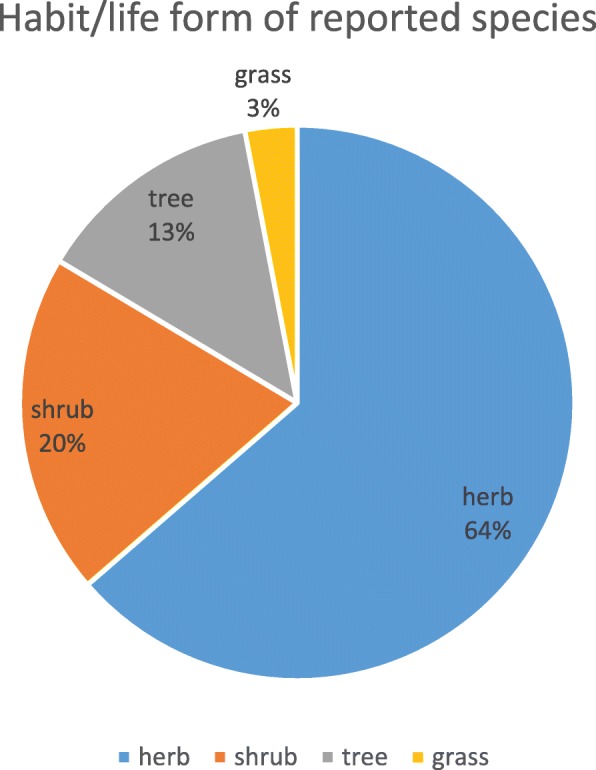
Details of the age and gender of participants of survey

**Fig. 6:**
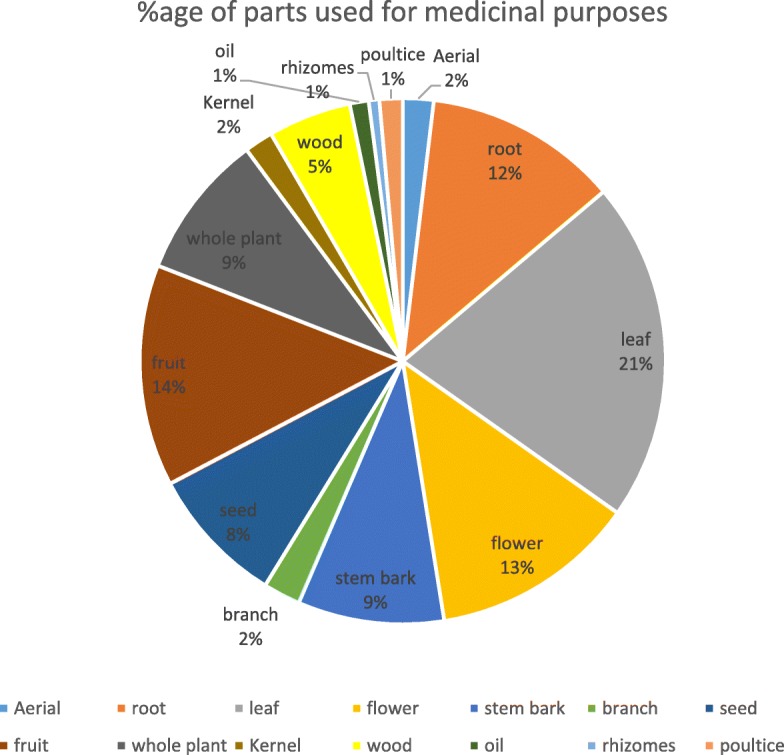
Age groups of participants, and the average number of species reported from different age group and retailers

We documented a total of 231 species representing 141 genera and 61 families (Table 2). *Compositae* was the most dominant family with 30 (12.9%) species reported, followed by Leguminosae, Lamiacceae, Rosaceae, Polygonaceae, Ranunculaceae, Salicaceae, Apiaceae, and Berberidaceae, with 16 (6.9%), 15 (6.5%), 15 (6.5%), 14 (6.1), 10 (4.3%), 10 (4.3%), 9 (3.9%), and 9 (3.9%) species, respectively. In total, 208 diseases were treated with documented species. The herbaceous flora accounts for 64% of the total reported species followed by shrubs at 20%, trees at 13%, and grasses at 3% (Fig. 5). Figure 6 represents the percentages of uses of different parts of the medicinal plants (further detail is available in Additional files 1, 2, 3, 4, 5, 6, 7, 8, 9, 10, 11, and 12 where a comprehensive list of species is provided besides the district level analysis).

During the survey, the participants revealed that medicinal plants are acquired from special locations and at a specific time, i.e., during a FGD in Upper Hunza (Gojal), the participants mentioned that medicinal plants are found both at the village and in pastures, yet the plants from pastures are used for medicinal purposes as those found in villages are not considered effective for medication. This needs to be scientifically verified from the field area, yet cases of same plant species presenting different chemical traits has been a known fact [94, 95].

### Occurrence and markets for medicinal plants in Gilgit-Baltistan

**Fig. 7:**
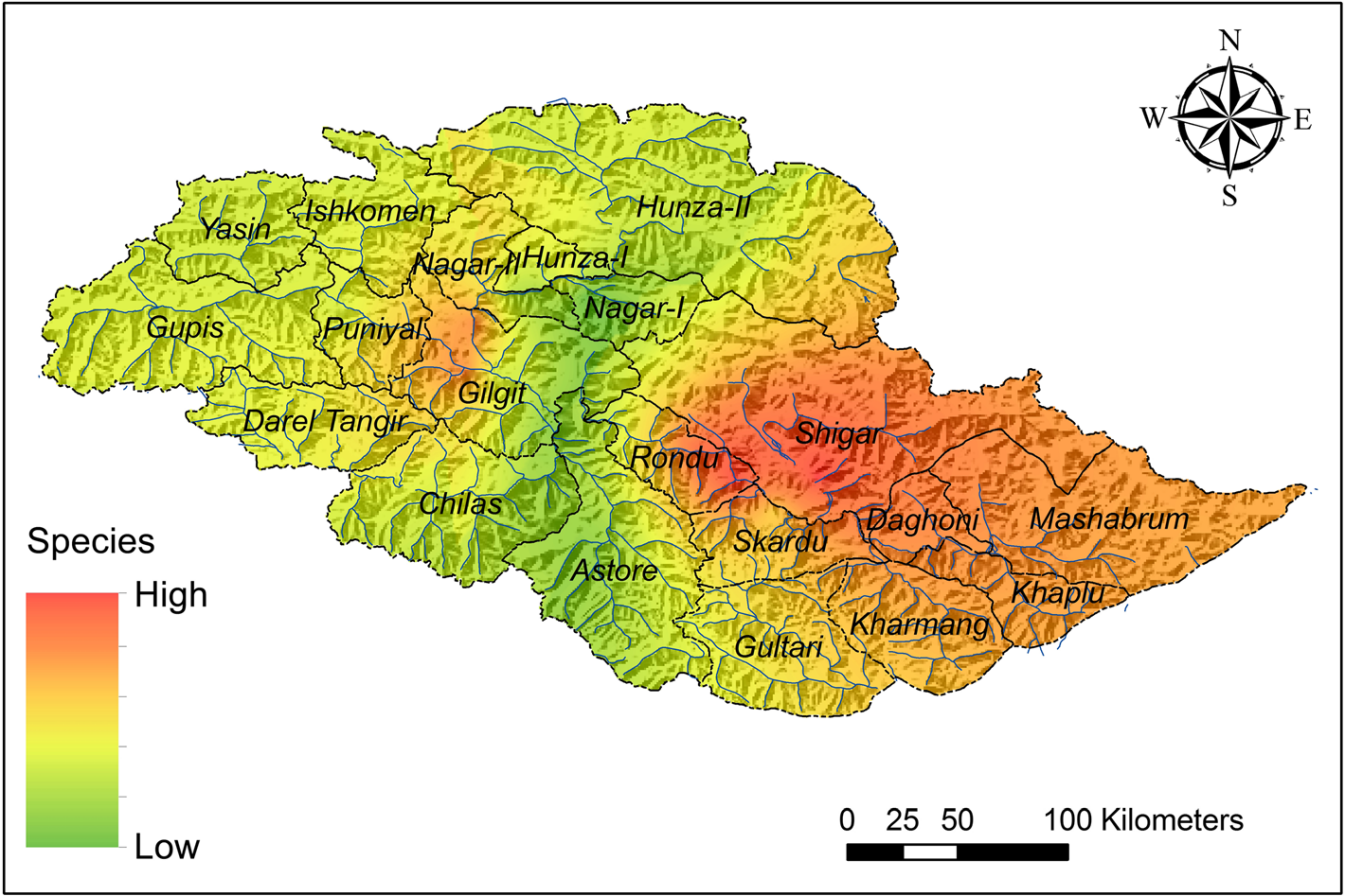
Habitat/life form of species reported during the survey

Interpolated results revealed that the utilization as well as high occurrence of medicinal plants is mainly concentrated in two locations (Fig. 7). Valleys from Skardu, Gilgit, and Ghizer are reported for the highest number of medicinal plants and their uses. There are no formal markets for medicinal plants in the region [35]. Gilgit and Skardu being the main business hubs serve as main markets for wholesale, retail, and purchase of medicinal plants. Besides, small amounts of the medicinal plants are supplied to bigger markets in Rawalpindi and Lahore by the wholesalers. The market players reported Skardu, Astore, and Gilgit districts as the main suppliers of medicinal plants as well.

Participants from the market revealed that most of the large herbal medicine production companies in Pakistan rely on raw materials from Indian Territory representing the same region across the border. Although a clear percentage of product flow was not known, during the market survey, the participants mentioned that the current markets for locally available plants are limited to the small town market centers and partly target markets in big cities like Rawalpindi (10%) and Lahore (10%) with meager shares moving out across the border to China (1–2%). The participants emphasized the involvement of government agencies for sustainable promotion, collection, and utilization of diverse plant resources in the region.

According to the retailers in Gilgit-Baltistan, *Thymus linearis*, *Delphinium brunonianum*, *Bergenia stracheyi*, *Saussurea heteromalla*, *Saussurea lappa*, *Carthamus tinctorius*, *Peganum harmala*, *Rheum emodi*, *Mentha longifolia*, *Mentha arvensis*, *Valeriana wallichii*, *Berberis lyceum*, and *Elaeagnus rhamnoides* account for the most demanded and utilized species. Although most of the customers were aware of the uses, the retailers had significant knowledge of the plant species they sold. Our survey results revealed that there is a large group of locals in Gilgit-Baltistan who rely on traditional medicine, yet these practices and the knowledge associated with them is rapidly depleting from the region. All the participants included in the survey had used medicinal plants for treating a medical condition at least once in their life. 79.5% of participants reported a transfer of knowledge from family elders. Out of these, 48% were above 50 years, 38% above 40 years while only 13% were below 40 years of age. 29.1% reported learning from community elders, of whom 64% were above 50 years, 32% above 40 years and only 3% below 40 years. 15.6% reported that they had acquired knowledge through interaction with other people, including THPs, plant collectors, and traders from other communities that include 87% above 50 years and 13% above 40 years. 20.1% were not aware of any treatments through medicinal plants which included only 4 and 26% participants above 50 and 40 years of age respectively while 70% of these participants fell below 40 years of age. Our results clearly indicate that the main source of knowledge transfer rests within the family. Yet only 13% of participants below the age of 40 confirmed that they had received such knowledge from within the family. Traditional healers in the community are experts in recognizing and collecting medicinal plants while other community members are not fully aware of the exact timing for collection. This is one of the main reasons why knowledge transfer is mainly through family elders as family secrets are not shared with outsiders. This allows the family to practice special medicinal remedies as well as retain a good image as regular suppliers to the wholesalers, retailers, and THPs in the region. The gradual expansion of trade and an increasing demand for medicinal plants in and outside the region has a positive impact on knowledge sharing. Most of the suppliers involved in the supply chain of medicinal plants represent the age group 40–60 which raises great concern for the future. Those representing the younger age group are either not collecting proper plants at the proper time or are too keen on gaining more financial benefits, thus not taking long-term conservation into account while collecting. This ever increasing gap between the young and old generation is affecting the knowledge transfer mechanism, in combination with over-exploitation and lack of conservation strategies, and the impacts of climatic changes. Such issues have also been observed in other studies [9, 30, 34, 42].

### Informant consensus factor (ICF)

The ICF analysis was done separately for each location in order to assess a clearer picture on which diseases stand out at each location and how many plant species are utilized for their treatment. Table 1 includes the ICF values for diseases divided into 29 disease categories from each of the study locations. The table indicates that stomach and intestinal disorders, respiratory disorders, skin infections, internal and external wounds, pain relief, ear, nose and throat disorders, hepatitis, and livestock diseases were the top disease categories reported from all the field sites during the field study. Most of the ailment categories were reported with a high ICF value based on the number of species and their usage reports. This is an indication of homogeneity in responses of the participants from each of the study sites in terms of the medicinal plant species and the modes of their use for addressing a particular disease. The results from ICF values clearly indicate that diseases related to stomach and intestinal disorders; respiratory disorders especially asthma; skin infections; and ear, nose, and throat infections were most common diseases, which is also supported by various publications [13, 18, 41]. Besides, the communities generally rely on medicinal plants for treating different kinds of internal and external wounds [10, 13, 18, 39, 41]. It is also evident that community members owning livestock hugely depend on traditional mode of medication [38].

**Table 1:** ICF, number of uses, and species used for each disease category

Category of diseases	Central Hunza					Ghizer					Gojal Hunza					Jalalabad					Kargha					Nagar					Skardu					Astore				
	ICF	Number of use reports	% age of use report	Number of species used	% age of species	ICF	Number of use reports	% age of use report	Number of species used	% age of species	ICF	Number of use reports	% age of use report	Number of species used	% age of species	ICF	Number of use reports	% age of use report	Number of species used	% age of species	ICF	Number of use reports	% age of use report	Number of species used	% age of species	ICF	Number of use reports	% age of use report	Number of species used	% age of species	ICF	Number of use reports	% age of use report	Number of species used	% age of species	ICF	Number of use reports	% age of use report	Number of species used	% age of species
Wounds	0.92	103	4.82	9	5.77	0.93	101	4.12	8	3.85	0.89	48	6.16	6	5.77	0.94	97	3.97	7	4.24	0.89	185	9.36	21	8.71	0.91	139	7.18	13	7.39	0.87	209	9.04	28	9.27	0.89	19	3.85	3	4.23
Skin infections	0.92	163	7.62	14	8.97	0.93	230	9.38	16	7.69	0.89	36	4.62	5	4.81	0.95	256	10.49	15	9.09	0.91	138	6.98	13	5.39	0.90	148	7.64	15	8.52	0.90	222	9.60	24	7.95	0.89	45	9.13	6	8.45
Pain relief	0.95	340	15.90	17	10.90	0.92	227	9.25	18	8.65	0.92	49	6.29	5	4.81	0.94	290	11.88	17	10.30	0.90	199	10.07	20	8.30	0.93	214	11.05	15	8.52	0.89	203	8.78	23	7.62	0.91	23	4.67	3	4.23
Kidney and uterus	0.95	101	4.72	6	3.85	0.90	99	4.04	11	5.29	0.94	19	2.44	2	1.92	0.96	122	5.00	6	3.64	0.86	110	5.56	16	6.64	0.95	118	6.10	7	3.98	0.85	137	5.92	21	6.95	0.89	38	7.71	5	7.04
Diaphoretic	0.00	0	0.00	0	0.00	1.00	7	0.29	1	0.48	0.00	0	0.00	0	0.00	0.00	0	0.00	0	0.00	1.00	3	0.15	1	0.41	0.00	0	0.00	0	0.00	0.00	0	0.00	0	0.00	0.00	0	0.00	0	0.00
Others	0.95	82	3.83	5	3.21	0.93	141	5.75	11	5.29	0.88	70	8.99	9	8.65	0.91	36	1.47	4	2.42	0.89	20	1.01	3	1.24	0.93	110	5.68	10	5.68	0.00	0	0.00	0	0.00	0.00	0	0.00	0	0.00
Cardiac stimulant	1.00	11	0.51	1	0.64	0.91	36	1.47	4	1.92	0.85	14	1.80	3	2.88	0.94	91	3.73	6	3.64	0.85	14	0.71	3	1.24	0.95	20	1.03	2	1.14	0.89	10	0.43	2	0.66	0.00	0	0.00	0	0.00
Stomach and intestine	0.94	305	14.26	18	11.54	0.93	429	17.49	30	14.42	0.90	126	16.17	14	13.46	0.96	236	9.67	11	6.67	0.90	483	24.43	51	21.16	0.91	271	14.00	24	13.64	0.90	638	27.58	63	20.86	0.87	107	21.70	15	21.13
Asthma/breathing/respiratory/pulmonary	0.93	180	8.42	14	8.97	0.94	261	10.64	17	8.17	0.90	80	10.27	9	8.65	0.94	134	5.49	9	5.45	0.90	145	7.33	15	6.22	0.93	122	6.30	9	5.11	0.86	210	9.08	30	9.93	0.90	68	13.79	8	11.27
Anti-inflammatory	0.92	14	0.65	2	1.28	0.90	62	2.53	7	3.37	0.92	14	1.80	2	1.92	0.91	65	2.66	7	4.24	0.84	51	2.58	9	3.73	0.90	41	2.12	5	2.84	0.83	42	1.82	8	2.65	0.00	0	0.00	0	0.00
Cancers	1.00	8	0.37	1	0.64	0.93	44	1.79	4	1.92	0.00	0	0.00	0	0.00	1.00	67	2.74	1	0.61	0.93	41	2.07	4	1.66	0.00	0	0.00	0	0.00	0.00	0	0.00	0	0.00	1.00	5	1.01	1	1.41
Cytoprotective	0.92	50	2.34	5	3.21	0.94	124	5.06	8	3.85	0.88	9	1.16	2	1.92	0.94	122	5.00	8	4.85	0.92	66	3.34	6	2.49	0.92	40	2.07	5	2.84	0.89	20	0.86	3	0.99	1.00	5	1.01	1	1.41
HIV	0.00	0	0.00	0	0.00	0.00	0	0.00	0	0.00	0.00	0	0.00	0	0.00	0.00	0	0.00	0	0.00	0.00	1	0.05	1	0.41	0.00	0	0.00	0	0.00	0.00	0	0.00	0	0.00	0.00	0	0.00	0	0.00
Hepatitis	0.90	94	4.39	10	6.41	0.90	50	2.04	6	2.88	0.85	34	4.36	6	5.77	0.92	66	2.70	6	3.64	0.78	38	1.92	9	3.73	0.92	65	3.36	6	3.41	0.85	49	2.12	8	2.65	0.85	41	8.32	7	9.86
Anti-stress/hypertension	0.93	70	3.27	6	3.85	0.93	42	1.71	4	1.92	0.85	14	1.80	3	2.88	0.95	39	1.60	3	1.82	0.87	16	0.81	3	1.24	0.92	40	2.07	4	2.27	0.88	33	1.43	5	1.66	1.00	14	2.84	1	1.41
Hepatoprotective/liver	0.94	19	0.89	2	1.28	0.92	27	1.10	3	1.44	0.00	0	0.00	0	0.00	0.91	83	3.40	8	4.85	0.86	23	1.16	4	1.66	0.96	24	1.24	2	1.14	0.81	28	1.21	6	1.99	1.00	4	0.81	1	1.41
Ear, nose, and throat	0.94	211	9.86	14	8.97	0.93	177	7.22	14	6.73	0.90	101	12.97	12	11.54	0.94	266	10.90	17	10.30	0.87	143	7.23	19	7.88	0.95	169	8.73	10	5.68	0.84	139	6.01	24	7.95	0.86	43	8.72	7	9.86
Menses/diseases	0.90	43	2.01	5	3.21	0.88	17	0.69	3	1.44	1.00	5	0.64	1	0.96	0.93	31	1.27	3	1.82	0.90	11	0.56	2	0.83	0.90	50	2.58	6	3.41	0.90	11	0.48	2	0.66	1.00	5	1.01	1	1.41
Brain and nervous disorders	1.00	10	0.47	1	0.64	0.89	28	1.14	4	1.92	0.00	0	0.00	0	0.00	0.93	128	5.24	10	6.06	1.00	5	0.25	1	0.41	0.81	17	0.88	4	2.27	0.86	8	0.35	2	0.66	0.00	0	0.00	0	0.00
Weight loss and fat reduction	0.96	26	1.22	2	1.28	0.86	23	0.94	4	1.92	0.90	11	1.41	2	1.92	0.00	0	0.00	0	0.00	1.00	7	0.35	1	0.41	0.91	45	2.32	5	2.84	0.89	20	0.86	3	0.99	0.00	0	0.00	0	0.00
Eye diseases	0.96	26	1.22	2	1.28	0.93	15	0.61	2	0.96	0.88	17	2.18	3	2.88	0.93	28	1.15	3	1.82	0.93	15	0.76	2	0.83	0.94	17	0.88	2	1.14	0.83	25	1.08	5	1.66	0.00	0	0.00	0	0.00
Diabetes	0.97	32	1.50	2	1.28	0.90	62	2.53	7	3.37	1.00	5	0.64	1	0.96	0.92	39	1.60	4	2.42	0.87	16	0.81	3	1.24	0.90	64	3.31	7	3.98	0.82	29	1.25	6	1.99	0.00	0	0.00	0	0.00
Teeth and gums	0.92	39	1.82	4	2.56	0.91	35	1.43	4	1.92	0.00	0	0.00	0	0.00	0.93	45	1.84	4	2.42	0.85	21	1.06	4	1.66	0.92	26	1.34	3	1.70	0.88	41	1.77	6	1.99	0.88	26	5.27	4	5.63
Blood purifier/diseases	0.93	84	3.93	7	4.49	0.91	131	5.34	13	6.25	0.91	23	2.95	3	2.88	0.95	111	4.55	7	4.24	0.86	64	3.24	10	4.15	0.93	69	3.56	6	3.41	0.87	125	5.40	17	5.63	0.87	31	6.29	5	7.04
Vomiting/nausea/altitude sickness	0.00	0	0.00	0	0.00	0.00	0	0.00	0	0.00	1.00	3	0.39	1	0.96	0.00	0	0.00	0	0.00	0.00	0	0.00	0	0.00	1.00	7	0.36	1	0.57	1.00	7	0.30	1	0.33	1.00	7	1.42	1	1.41
Livestock diseases	0.96	94	4.39	5	3.21	0.92	61	2.49	6	2.88	0.87	76	9.76	11	10.58	0.93	44	1.80	4	2.42	0.89	132	6.68	15	6.22	0.88	75	3.87	10	5.68	0.87	102	4.41	14	4.64	0.91	12	2.43	2	2.82
Sexual diseases/stimulant	1.00	9	0.42	1	0.64	0.94	17	0.69	2	0.96	0.94	19	2.44	2	1.92	0.94	19	0.78	2	1.21	1.00	5	0.25	1	0.41	1.00	4	0.21	1	0.57	0.00	0	0.00	0	0.00	0.00	0	0.00	0	0.00
Hemorrhoids/piles	1.00	7	0.33	1	0.64	1.00	7	0.29	1	0.48	1.00	3	0.39	1	0.96	0.92	14	0.57	2	1.21	0.88	25	1.26	4	1.66	0.94	33	1.70	3	1.70	1.00	5	0.22	1	0.33	0.00	0	0.00	0	0.00
Maternal health	0.94	18	0.84	2	1.28	0.00	0	0.00	0	0.00	1.00	3	0.39	1	0.96	1.00	12	0.49	1	0.61	0.00	0	0.00	0	0.00	1.00	8	0.41	1	0.57	0.00	0	0.00	0	0.00	0.00	0	0.00	0	0.00

### Relative frequency of citation (RFC) and use value (UV)

The RFC and UV values for each plant species were calculated in order to validate the frequency of citation for the species used for different ailment categories. The values were calculated at district level in order to authenticate the local frequency of use. The RFC value is used for verifying the use of a medicinal plant species for different diseases while the UV value is an indication for the relative importance of these species in a particular population [78]. The highest RFC value from all the sites was calculated for *Dracocephalum nuristanicum* Rech.f. & Edelb. (0.7) followed by *Cupressus sempervirens* L., *Prunella vulgaris* L., and *Potentilla argyrophylla* Wall. ex Lehm., averaged at 0.47 each indicating that these species were highly reported by the participants of the study. RFC directly depends on the number of participants mentioning use of a particular plant (FC); therefore, the abovementioned plants were very commonly used in the study area. The UV values for *Amaranthus viridis* L. (0.33), *Artemisia herba-alba* Asso (0.28) and *Astragalus zanskarensis* Bunge (0.25), and *Aconitum violaceum* Jacquem. Ex. Stapf (0.25) were the highest averaged from the field sites. The results indicate the usage and reliance on medicinal plants for treatment of multiple diseases. Such reliance on medicinal plants in both humans and livestock are reported from the region [9, 16, 18, 30, 34, 38, 41]. Table 2 includes a list of reported species from all the six districts of Gilgit-Baltistan with their local names, parts used, mode of their utilization, the average values for RFC and UV, number of ailment categories addressed, and the number of uses reported for each of the plant species. Area wise details for each of these species is provided in Additional files 2, 3, 4, 5, 6, 7, 8, and 9 while the detailed list of diseases categorized in 29 ailment categories is provided in S12.

**Table 2:** RFC, UV, number of uses, and ailments of species from each location

S no.	Family	Species	Local name	Part used (Kargha-K, Nagar-N, Skardu-S, Ghizer-Gh, Hunza-H, Astore-A, Gilgit-G)	Mode of use (Kargha-K, Nagar-N, Skardu-S, Ghizer-Gh, Hunza-H, Astore-A, Gilgit-G)	Location (Astore-Astore, Ghizer-Ghizer, Gojal-Gojal, Central Hunza-Hunza, Jalalabad-Jalalabad, Kargha-Kargha, Nagar-Nagar, Skardu-Skardu)	Average of RFC	Average of UV	Ailment categories	No. of use responses	Voucher no.	Previous citation	
1	Amaranthaceae	*Aerva lanata* (L.) Juss.	Shutpask	Whole plant	Ash (H, N, K, Gh, A), decoction (S, A, Gh, H, K)	Gojal	0.175	0.214286	3	14	MAS-089	17	
2		*Achyranthes aspera* L.	larghaky	Flower	Paste	Kargha	0.2	0.176471	3	17	MAS-238	40, 41	
3		*Chenopodium album* L.	Snew, Sheleet, Kunoaw	Leaf (S), whole plant (H, Gh	Paste, infusion, poultice, decoction	Ghizer, Gojal, Hunza, Skardu	0.21875	0.136706	8	59	MAS-121, MAS-153, MAS-457	9, 12, 13, 17, 33, 37	
4		*Dysphania botrys* (L.) Mosyakin & Clemants	Khama, Khord	Aerial (K), whole plant (S, H)	Powder, decoction	Gojal, Jalalabad, Skardu	0.194444	0.12619	7	57	MAS-128, MAS-229, MAS-482	35	
5		*Amaranthus viridis* L.	Dhimdo	Leaf	Paste, direct	Kargha, Nagar	0.1	0.333333	3	10	MAS-314, MAS-376	14	
6		*Allium humile* Kunth	Chung	Bulb	Infusion, direct	Skardu	0.266667	0.176471	3	17	MAS-381	12, 13, 17	
7		*Allium carolinianum* DC.	Kachpauk, Booma, Chong	Bulb (K, S, A), leaf, bulb (H)	Paste (S), direct, decoction	Astore, Gojal, Kargha, Nagar, Skardu	0.205	0.22988	21	102	MAS-012, MAS-112, MAS-346, MAS-424	12, 13, 17	
8		*Allium cepa* L.	Ghashoo, Xong, Song	Bulb (K), leaf, bulb (S), poultice, bulb (H)	Poultice, decoction, direct (H, S, Gh, N, K), paste (S, A, K, Gh)	Hunza, Kargha, Nagar, Skardu	0.16875	0.202381	9	50	MAS-179, MAS-305, MAS-365, MAS-489	9, 33, 38, 39	
9		*Allium sativum* L.	Zgoqpa, Bukpa	Bulb	Direct	Hunza, Kargha, Nagar, Skardu	0.31875	0.110367	11	149	MAS-180, MAS-306, MAS-366, MAS-490	9, 33, 39	
10	Anacardiaceae	*Pistacia mutica* Fisch. & C.A.Mey.	Daraaw	Branches	Oil	Hunza	0.2	0.142857	1	7	MAS-137	9, 33	
11		*Pistacia khinjuk* stocks	Kakavomn	Galls, resin, wood, leaf	Direct, decoction	Jalalabad	0.175	0.071429	6	84	MAS-203	15	
12	Apiaceae	*Heracleum candicans* Wall. ex DC.	Ghang	Leaf	Decoction	Skardu	0.266667	0.071429	1	14	MAS-392	12	
13		*Pimpinella diversifolia* DC.	Kohniod	Whole plant	Powder, decoction	Astore, Kargha, Skardu	0.269444	0.15	9	60	MAS-028, MAS-270, MAS-440	13	
14		*Pleurospermum candollei* (DC.) C.B. Clarke in Hook. f.	Braqshundun	Whole plant	Decoction	Astore, Skardu	0.35	0.142857	2	14	MAS-037, MAS-449	13	
15		*Angelica glauca* Edgew	Choro, Chora	Root (K), stem, seed, root (Gh)	Decoction, powder, direct	Ghizer, Kargha	0.216667	0.166667	10	55	MAS-078, MAS-284	11, 16	
16		*Carum carvi* L.	Filizooh, Zera, Hayyo	Seed (K, S), seed, fruit (Gh)	Decoction, powder, direct (Gh)	Ghizer, Kargha, Skardu	0.205556	0.139184	8	66	MAS-084, MAS-290, MAS-472	11, 12, 16	
17		*Daucus carota* L.	Phopuce, Jangli, Jut Ghachoon, Ghasoon, Gholafuvi lona	Leaf, seed (H, N), leaf (S)	Direct, decoction	Hunza, Kargha, Nagar, Skardu	0.291667	0.134921	7	57	MAS-181, MAS-307, MAS-367, MAS-491	9, 32, 33	
18		*Coriandrum sativum* L.	Ausu, Naski	Seed	Decoction, direct	Hunza, Kargha, Skardu	0.213889	0.119929	4	41	MAS-183, MAS-309, MAS-493	9, 33, 39	
19		*Foeniculum vulgare* Mill.	Badian	Fruit (K), seed (S)	Decoction	Kargha, Skardu	0.2375	0.099206	2	25	MAS-326, MAS-511	38, 39	
20		*Heracleum pinnatum* C.B. Clarke	Hltireet	Leaf	Direct	Kargha, Skardu	0.295833	0.142857	2	14	MAS-328, MAS-513	39	
21	Asteraceae	*Allardia tomentosa* Decne.	Tarkham	Leaf, flower	Grinded	Skardu	0.2	0.230769	3	13	MAS-380	39	
22		*Leontopodium leontopodinum*	Naqposhoto	Seed	Decoction	Kargha, Skardu	0.341667	0.098086	4	41	MAS-329, MAS-514	39	
23	Berberidaceae	*Podophyllum emodi* Wall. ex Hook.f. & Thomson	Shingoy	Root, rhizome	Decoction	Kargha	0.2	0.2	2	10	MAS-245	11, 53	
24		*Berberis pseudumbellata* R.Parker	Ishkeen, Shokurum, Skyurboo	Root, stem, bark (K), whole plant (S), flower, fruit, seed (A)	Decoction, powder	Astore, Jalalabad, Kargha, Skardu	0.325	0.114213	10	111	MAS-019, MAS-220, MAS-261, MAS-431	13, 15, 19, 27, 34, 39	
25		*Berberis lycium* Royle	Zolg, Ishkeen, Skyurboo	Root, leaf, seed, bark, fruit, flower (S, K, N), root, leaf, fruit (H), root, leaf, stem, fruit (Gh)	Decoction	Ghizer, Gojal, Hunza, Kargha, Nagar, Skardu	0.322222	0.092532	55	743	MAS-061, MAS-118, MAS-351, MAS-454	9, 14, 16, 17, 32, 33, 34, 37, 38	
26		*Berberis brandisiana* Ahrendt	Ishkeen, Ishkenachi	Root, stem, bark	Decoction	Jalalabad, Kargha, Nagar, Skardu	0.33125	0.114998	39	490	MAS-235, MAS-312, MAS-374, MAS-497	15, 34	
27		*Berberis orthobotrys* Bien. ex Aitch.	Ishkeen,Skyurboo	Root, stem, bark (K), root, stem (S	Decoction	Jalalabad, Kargha, Skardu	0.341667	0.112444	15	121	MAS-236, MAS-313, MAS-498	15, 34	
28		*Berberis parkeriana* C.K.Schneid.	Ishkeen,Skyurboo	Root, stem	Decoction	Kargha, Skardu	0.354167	0.154762	5	33	MAS-317, MAS-502	34	
29		*Berberis stewartiana* Jafri	Ishkeen, Shokurum	Root, leaf	Decoction	Kargha, Skardu	0.25	0.224599	6	28	MAS-318, MAS-503	34	
30		*Berberis ulicina* Hook.f. & Thomson	Ishkeen, Shokurum	Root, leaf	Decoction	Kargha, Skardu	0.2625	0.204861	5	25	MAS-319, MAS-504	34	
31		*Berberis vulgaris* L.	Ishkeen, Shokurum	Fruit (S), leaf, fruit (K)	Decoction, direct	Kargha, Skardu	0.304167	0.154762	6	39	MAS-320, MAS-505	12, 34, 39	
32	Betulaceae	*Betula utilis* D.Don	Xuxi, Halli, Jowzee, Furze, Staqpa	Bark, wood (N, K, H), bark (S)	Decoction, direct	Gojal, Hunza, Jalalabad, Kargha, Nagar, Skardu	0.2375	0.099554	13	143	MAS-123, MAS-167, MAS-225, MAS-477	9, 11, 13, 15, 17, 39, 53	
33	Boraginaceae	*Onosma hispida* Wall. ex G. Don	Kangmar	Whole plant	Decoction	Astore, Kargha	0.291667	0.162338	5	32	MAS-020, MAS-262	11, 13	
34	Brassicaceae	*Brassica oleracea var. botrytis* L.	Phul Gobi	Flower	Direct	Hunza	0.175	0.166667	1	6	MAS-132	9	
35		*Descurainia sophia* (L.) Webb ex Prantl	Khashir	Whole plant	Powder, decoction	Astore, Skardu	0.233333	0.174242	4	23	MAS-034, MAS-446	13	
36		*Raphanus sativus* L.	Moolo, Gholafuvi sonma	Leaf	Direct	Hunza, Kargha, Nagar, Skardu	0.2875	0.071584	5	99	MAS-182, MAS-308, MAS-368, MAS-492	9, 39	
37		*Brassica oleracea var. capitata* L.	Band Gobi	Flower	Direct	Hunza, Nagar	0.15	0.133333	2	16	MAS-186, MAS-369	9	
38		*Brassica juncea* (L.) Czern.	Sarsung mar	Seed	Oil	Kargha, Skardu	0.191667	0.142857	2	14	MAS-323, MAS-508	39	
39		*Lepidium latifolium* L.	Sonma	Leaf (K), leaf, root (S)	Powder, infusion (S)	Kargha, Skardu	0.3375	0.171429	4	22	MAS-330, MAS-515	12	
40	Buxaceae	*Buxus papillosa* C.K. Schneid	Angaroo	Leaf	Oil	Skardu	0.233333	0.083333	1	12	MAS-388	38	
41	Campanulaceae	*Codonopsis clematidea* (Schrenk) C.B.Clarke	Loo sunma/Bajo mindoq	Flower		Astore, Skardu	0.366667	0.107143	2	21	MAS-032, MAS-444	12, 13	
42	Cannabinaeae	*Cannabis sativa* L.	Thoonch	Seed (N, H), whole plant (K)	Direct	Hunza, Kargha, Nagar	0.258333	0.132762	12	101	MAS-178, MAS-304, MAS-364	9, 14, 33, 53	
43	Capparaceae	*Capparis spinosa* L.	Kraba, Kavir, Kappar, Chopir, Shorot, Champarrang, Thoonch	Root, bark, fruit, seed, branches, flower (N, K), root, fruit, seed (S), seed, flower, fruit (H)	Oil (H, N, K), powder, decoction, oil (S), paste (H)	Gojal, Hunza, Jalalabad, Kargha, Nagar, Skardu	0.276389	0.093599	25	302	MAS-124, MAS-226, MAS-359, MAS-478	9, 12, 15, 17, 33, 37, 38, 39, 53	
44	Caprifoliaceae	*Lonicera microphylla* Willd. ex Schult.	Pushkar	Stem, brnaches, fruit	Paste	Jalalabad	0.2	0.043478	1	23	MAS-199	15	
45		*Valeriana wallichii* DC.	Mushk-bala	Root (K, H), rhizomes (S)	Powder, decoction, paste	Gojal, Kargha, Skardu	0.266667	0.149335	6	40	MAS-130, MAS-300, MAS-484	11, 17, 38	
46		*Lonicera asperifolia* Hook. f. & Thomson	Krraba	Leaf	Direct	Kargha, Skardu	0.270833	0.142857	2	14	MAS-331, MAS-516	39	
47	Caryophyllaceae	*Cerastium fontanum* Baumg.	Bloghar	Whole plant	Direct	Astore, Kargha, Skardu	0.222222	0.118276	6	54	MAS-024, MAS-266, MAS-436	13	
48	Compositae	*Anaphalis nepalensis*	Chikee	Flower, fruit	Dried flower, powder, fume (Gh)	Ghizer	0.233333	0.142857	1	7	MAS-039	16	
49		*Artemisia annua* L.	Xoon	Whole plant	Direct	Ghizer	0.233333	0.157895	3	19	MAS-040	37	
50		*Artemisia dubia* Wall. Ex Bess.	Bursay	Whole plant	Paste, powder	Ghizer	0.2	0.142857	3	21	MAS-041	37	
51		*Artemisia herba-alba* Asso	Kho Bursay	Whole plant	Decoction	Ghizer	0.2	0.272727	3	11	MAS-042	37	
52		*Saussurea heteromalla* (D.Don) Hand.-Mazz	Kali zira	Weed	Paste, direct	Ghizer	0.1	0.111111	2	18	MAS-049	16	
53		*Anaphalis triplinervis* (Sims) Sims ex C.B.Clarke	Yeepwoosh	Leaf, flower	Poultice, dried leaf and flower	Gojal	0.225	0.166667	4	24	MAS-090	17	
54		*Tragopogon dubius* Scop	Kreel woosh	Flower	Decoction	Gojal	0.15	0.142857	1	7	MAS-104	17	
55		*Achillea millefolium* L.	Yarrow	Flower	Decoction, poultice	Kargha	0.175	0.142857	3	21	MAS-237	40, 41	
56		*Artemisia laciniata* Willd.	Khampa	Leaf	Paste	Kargha	0.3	0.142857	1	7	MAS-239	11, 12	
57		*Artemisia rutifolia* Spreng. Ex Spreng	Kho Bursay	Aerial	Paste	Kargha	0.175	0.142857	1	7	MAS-240	38	
58		*Artemisia fragrans* Willd	Kho Bursay	Aerial	Powder	Skardu	0.266667	0.166667	1	6	MAS-384	38	
59		*Artemisia santolinifolia* Turcz. Ex Krasch.	Kho Bursay	Leaf, stem	Powder, paste	Skardu	0.266667	0.2	2	10	MAS-385	39	
60		*Jurinea dolomiaea* Boiss.	Sathing	Leaf, root	Decoction, poultice	Skardu	0.233333	0.214286	3	14	MAS-393	12	
61		*Pseudognaphalium luteoalbum* (L.) Hilliard & B. L. Burtt	Thliri	Leaf	Decoction	Skardu	0.366667	0.142857	1	7	MAS-404	12	
62		*Senecio chrysanthemoides* DC.	Api mindoq	Leaf, flower, root	Decoction, poultice	Skardu	0.233333	0.157895	3	19	MAS-409	12	
63		*Tanacetum senecionis* (Jacquem. ex Besser) J.Gay ex DC.	Hilteree/Tialo	Flower	Powder, infusion, decoction	Skardu	0.4	0.166667	3	18	MAS-411	38	
64		*Taraxacum officinale* (L.) Weber ex F.H.Wigg.	Doduli, Mamo Shikinachi, Ishkanachi, Shantha, Talkhting, Khosmas	Leaf, root (K, Gh), leaf, flower (S), leaf, latex (H), latex (A)	Decoction, powder (K), infusion (S)	Astore, Ghizer, Gojal, Hunza, Kargha, Skardu	0.222222	0.161198	18	136	MAS-003, MAS-051, MAS-415	9, 13, 14, 16, 17, 33, 37, 38, 53	
65		*Artemisia brevifolia* Wall	Rooner, Bursay, Taroqtpesk, Bustae	Leaf (N, K), leaf, flower (S, H), whole plant (A,Gh)	Poultice, direct	Astore, Ghizer, Gojal, Kargha, Nagar, Skardu	0.216667	0.218759	12	62	MAS-006, MAS-054, MAS-109, MAS-418	13, 17, 35, 37, 38, 39	
66		*Artemisia maritima* L.	Rooner, Zoon, Bursay	Leaf, bud, flower (N, K, Gh), aerial (H), flower (A)	Direct, paste, decoction (Gh)	Astore, Ghizer, Hunza, Kargha, Nagar, Skardu	0.270833	0.112619	18	228	MAS-007, MAS-055, MAS-142, MAS-419	9, 11, 14, 16, 33, 37, 39, 40, 41, 53	
67		*Artemisia sieversiana* Ehrh.	Hampa, Khampa	Leaf (K, A, N), leaf, flower, root (S)	Infusion, decoction, paste	Astore, Kargha, Nagar, Skardu	0.183333	0.20211	6	29	MAS-022, MAS-264, MAS-349, MAS-434	12, 39	
68		*Cousinia thomsonii* C.B.Clarke	Charchu	Flower	Diret	Astore, Skardu	0.266667	0.142857	2	14	MAS-033, MAS-445	13	
69		*Carthamus tinctorius* L.	Pock, Poong	Flower, seed	Decoction, oil	Ghizer, Hunza	0.370833	0.079828	14	175	MAS-066, MAS-154, MAS-459	9, 16, 32, 33, 37	
70		*Echinops echinatus* Roxb.	Jacheer	Whole plant	Decoction, direct	Ghizer, Hunza, Kargha, Nagar	0.260417	0.103175	6	96	MAS-072, MAS-160, MAS-281, MAS-356	9, 14, 16	
71		*Saussurea lappa* (Decne.) Sch.Bip.	Minal	Root (K), stem, root (Gh)	Powder, paste, decoction	Ghizer, Kargha	0.204167	0.139959	9	75	MAS-082, MAS-288	11, 16, 37, 53	
72		*Artemisia scoparia* Waldst. & Kitam.	Khobustae	Leaf, flower (K, S), whole plant (Gh)	Paste, fume (Gh), decoction (S)	Ghizer, Kargha, Skardu	0.202778	0.176667	6	43	MAS-083, MAS-289, MAS-471	13, 37	
73		*Cichorium intybus* L.	Ishkinachi, Caroop, qarali Chicknachi	Whole plant (N, K), root, leaf (S)	Direct, infusion, decoction, decoction (H)	Gojal, Hunza, Kargha, Nagar, Skardu	0.251667	0.148798	12	99	MAS-126, MAS-297, MAS-360, MAS-480	12, 14, 17, 33	
74		*Tanacetum gracile* Hook.f. & Thomson	Cerpho bursay, serfo bursay, Bursay	Leaf	Decoction, powder (S), direct	Hunza, Kargha, Skardu	0.252778	0.155844	5	32	MAS-185, MAS-311, MAS-495	12, 39, 40, 41	
75		*Artemisia absinthium* L.	Zoon	Whole plant	Infusion, paste, powder	Kargha, Nagar	0.175	0.177778	4	24	MAS-315, MAS-377	9, 11, 14	
76		*Seriphidium brevifolium* (Wall. ex DC.) Ling & Y.R.Ling	Bursay	Leaf (K), flower (S)	Powder, decoction, direct	Kargha, Skardu	0.320833	0.142857	2	14	MAS-336, MAS-521	12, 39	
77		*Tanacetum falconeri* Hook.f.	Haltiry, Htialo, Pholing, Zoon, Tyalo	Whole plant (K), leaf (S)	Direct, powder	Kargha, Skardu	0.204167	0.190909	4	21	MAS-338, MAS-523	13, 39	
78	Convolvulaceae	*Canvolvulus arvensis* L.	Thringthringmo	Whole plant	Decoction, powder	Astore, Kargha, Skardu	0.197222	0.166667	3	18	MAS-023, MAS-265, MAS-435	13	
79		*Cuscuta reflexa* Roxb.	Ghbul thaq	Stem, flower (K), whole plant (S)	Decoction, direct (S)	Kargha, Skardu	0.2375	0.1625	4	26	MAS-325, MAS-510	38, 39	
80	Crassulaceae	*Rhodiola imbricata* Edgew.	Chundol	Root	Powder	Skardu	0.233333	0.222222	4	18	MAS-406	12	
81	Cucurbitaceae	*Cupressus sempervirens* L*.*	Saro	Fruit, stem	Decoction, direct	Ghizer	0.466667	0.095238	2	21	MAS-044	16, 37	
82		*Citrullus vulgaris* Schrad.	Bowar	Fruit	Decoction	Hunza	0.225	0.045455	1	22	MAS-133	9, 33	
83		*Cucurbita pepo* L.	Hosar	Seed	Oil, direct	Nagar	0.4	0.085714	3	35	MAS-339	9	
84	Cupressaceae	*Juniperus squamata* Buch.	Cheleh, Chili, Hlashuk, Yarz, Shukpa	Fruit, twigs	Infusion, oil, paste	Ghizer	0.233333	0.132075	7	53	MAS-045	37	
85		*Juniperus turkestanica* Kom.	Cheleh	Leaf, fruit, wood	Powder, decoction	Jalalabad	0.175	0.054054	2	37	MAS-198	15	
86		*Juniperus excelsa* M.Bieb.	Cheleh, Chili, Hlashuk, Yarz, Shukpa	Fruit, wood, leaf (N, K, H), fruit (S, A)	Ash, powder, decoction, paste (Gh)	Astore, Ghizer, Gojal, Jalalabad, Kargha-Kargha, Nagar, Skardu	0.245238	0.157465	25	194	MAS-004, MAS-052, MAS-107, MAS-416	12, 13, 14, 15, 16, 17, 37	
87		*Juniperus communis* L.	Mitthary, Oshuk	Fruit, wood (K, A, S), fruit, wood, oil (Gh)	Infusion, decoction, paste, powder (A)	Astore, Ghizer, Jalalabad, Skardu	0.272917	0.145114	13	112	MAS-009, MAS-057, MAS-215, MAS-421	13, 15, 37	
88	Elaeagnaceae	*Elaeagnus rhamnoides* (L.) A.Nelson	Buru, Buroh, Seabuckthorn, Soq, Rema, Zakh, Chanso, Karsoq	Leaf, fruit, seed, root, wood (K, N), leaf, fruit, seed (S), fruit (H), fruit, stem, leaf (Gh), fruit, leaf (A)	Ash, direct, decoction, powder	Astore, Ghizer, Gojal, Hunza, Jalalabad, Kargha, Nagar, Skardu	0.323958	0.106938	43	747	MAS-002, MAS-050, MAS-105, MAS-414	9, 11, 12, 13, 14, 15, 17, 30, 33, 38, 39, 53	
89		*Elaeagnus angustifolia* L.	Shekarkuch, Gindawar, Ghonair, Sisk, Ghundair	Whole plant (N, K), flower, fruit, gum (Gh, H)	Direct, powder, decoction,	Ghizer, Gojal, Hunza, Jalalabad, Kargha, Nagar	0.295833	0.080555	25	331	MAS-059, MAS-116, MAS-350, MAS-452	9, 14, 15, 16, 17, 32, 33, 37	
90	Ephedraceae	*Ephedra intermedia* Schrenk & C.A.Mey.	Shaay Soom	Stem, root	Decoction	Jalalabad	0.2	0.061538	4	65	MAS-194	57	
91		*Ephedra gerardiana* Wall. ex Stapf	Soom, Say, Yemook, Sopat, Sopt	Whole plant (K, Gh), aerial (S), leaf, stem (H)	Decoction	Ghizer, Gojal, Hunza, Jalalabad, Kargha, Skardu	0.294444	0.097941	22	239	MAS-060, MAS-275, MAS-453	9, 11, 12, 14, 15, 16, 32, 33, 37, 38, 53	
92	Equisetaceae	*Equisetum arvense* L.	Thangshingy harswa, Thangshing stwa	Aerial (S), whole plant (A)	Decoction	Astore, Skardu	0.283333	0.142857	2	14	MAS-035, MAS-447	13, 38	
93	Ericaceae	*Rhododendron anthopogon* D. Don	Chauman	Leaf, flower	Infusion, decoction	Skardu	0.3	0.15	3	20	MAS-407	12	
94	Gentianaceae	*Swertia petiolata* D. Don	Brama	Leaf, root	Paste, decoction, powder (A)	Skardu	0.233333	0.210526	4	19	MAS-410	12	
95		*Gentiana olivieri* Griseb.	Tikta	Leaf, flower (K), leaf (S)	Direct	Kargha, Skardu	0.233333	0.162338	3	18	MAS-327, MAS-512	12, 39	
96	Geraniaceae	*Geranium nepalense* Sweet	Bamik	Fruit, root	Poultice, decoction, powder	Skardu	0.3	0.235294	4	17	MAS-391	12	
97	Grossulariaceae	*Ribes himalense* Royle ex Decne.	Murshatooh	Fruit	Powder	Jalalabad	0.175	0.075	3	40	MAS-204	15	
98		*Ribes orientale* Desf.	Ghonashatooh	Root	Powder	Jalalabad	0.2	0.033333	2	60	MAS-205	15	
99		*Ribes alpestre* Wall. ex Decne.	Shumlooh, Skioruru	Root, flower	Powder, direct	Astore, Gojal, Jalalabad, Skardu	0.189583	0.109821	9	109	MAS-011, MAS-111, MAS-217, MAS-423	13, 15, 17	
100	Iridaceae	*Crocus sativus L.*	Zafran	Flower	Powder	Kargha, Skardu	0.266667	0.142857	2	14	MAS-324, MAS-509	39	
101	Juglandaceae	*Juglans regia L.*	Achow, Ashooh	Root, kernel, wood (N), root, kernel, seed, wood (K), kernel (S, H)	Oil, direct	Hunza, Jalalabad, Kargha, Nagar, Skardu	0.276667	0.098536	11	139	MAS-173, MAS-301, MAS-488	9, 13, 15, 33	
102	Lamiaceae	*Nepeta floccosa* Benth.	Buzlanj	Leaf, flower	Decoction, infusion	Gojal	0.2	0.172414	5	29	MAS-098	17	
103		*Isodon rugosus* (Wall. ex Benth.) Codd	Phaypush	Leaf, branches	Powder	Jalalabad	0.2	0.097561	4	41	MAS-197	15	
104		*Dracocephalum nuristanicum* Rech.f. & Edelb.	Shamdun	Leaf, flower, seed	Paste, decoction, infusion	Skardu	0.7	0.064935	5	77	MAS-389	12, 38, 39	
105		*Mentha haplocalyx* Briq.	Shoma	Leaf	Direct, paste	Skardu	0.3	0.214286	3	14	MAS-394	39	
106		*Nepeta leucolaena* Benth. ex Hook.f.	Askuta	Whole plant	Powder, decoction	Skardu	0.366667	0.035714	1	28	MAS-395	12, 38	
107		*Prunella vulgaris* L.	Harswa	Leaf	Decoction	Skardu	0.466667	0.153846	4	26	MAS-403	12	
108		*Mentha royleana* Wall. ex Benth.	Foling, Gudunj	Leaf (K, A, S), leaf, flower (H)	Powder, paste (H), direct, infusion	Astore, Gojal, Kargha, Skardu	0.291667	0.104499	9	91	MAS-013, MAS-113, MAS-257, MAS-425	12, 13, 17, 38, 39	
109		*Thymus linearis* Benth.	Tumuro, Tumburu, Tumburuk	Whole plant (N, S), leaf, flower (H), flower (A)	Decoction, infusion	Astore, Gojal, Nagar, Skardu	0.222917	0.100514	12	151	MAS-014, MAS-114, MAS-347, MAS-426	12, 13, 17, 53	
110		*Mentha longifolia* (L.) L.	Fileel, Whadan, Phileel	Leaf, flower (H, K, N), leaf (Gh)	Powder, paste, decoction (Gh)	Ghizer, Gojal, Kargha, Nagar	0.252083	0.12514	29	319	MAS-065, MAS-122, MAS-278, MAS-353	14, 16, 17	
111		*Mentha sylvestris* L.	Bundoo	Leaf, flower	Decoction	Ghizer, Hunza	0.241667	0.101515	8	74	MAS-067, MAS-155, MAS-460	9, 33, 37, 53	
112		*Thymus serphyllum* L.	Tumuro, Ree tumburuk	Whole plant (K, Gh), leaf, flower (S), aerial (H)	Decoction	Ghizer, Hunza, Kargha, Skardu	0.33125	0.079512	17	268	MAS-074, MAS-162, MAS-283, MAS-467	9, 11, 14, 16, 33, 37, 38	
113		*Mentha arvensis* L.	Peeno	Leaf (S, K), whole plant (Gh)	Powder, direct, paste	Ghizer, Kargha, Skardu	0.275	0.138889	6	44	MAS-085, MAS-291, MAS-473	37, 38, 39	
114		*Perovskia abrotanoides* Kar.	Faring bursay	Flower	Infusion	Hunza, Kargha, Skardu	0.202778	0.126246	6	57	MAS-184, MAS-310, MAS-494	12, 39, 40, 41	
115		*Mentha spicata* L.	Podina	Whole plant	Decoction	Hunza, Nagar	0.3375	0.046801	8	160	MAS-189, MAS-372	9, 33	
116		*Stachys tibetica* Vatke	Khampa	Leaf	Powder, direct	Kargha, Skardu	0.304167	0.049043	2	41	MAS-337, MAS-522	39	
117	Leguminosae	*Astragalus frigidus* (L.) A.Gray	Shashal	Leaf, stem	Powder	Astore	0.233333	0.176471	3	17	MAS-001	13	
118		*Astragalus falconeri* Bunge	Hapocho	Leaf, stem	Powder	Ghizer	0.233333	0.083333	1	12	MAS-043	16	
119		*Melilotus officinalis* (L.) Pall.	Bissasing	Whole plant	Decoction	Ghizer	0.333333	0.115385	6	52	MAS-046	16, 37	
120		*Astragalus strictus* Benth.	Zhop/Thope	Leaf, flower	Direct	Gojal	0.175	0.142857	1	7	MAS-091	17	
121		*Melilotus alba* Ledeb.	Sinjhi	Aerial	Paste	Gojal	0.175	0.166667	2	12	MAS-096	17	
122		*Caragana brevifolia* Kom.	Hapoocho	Root	Direct, decoction	Jalalabad	0.175	0.058824	1	17	MAS-191	15	
123		*Caragana tragacanthoides* var. himalaica Komarov	Hapoocho	Root	Direct, decoction	Jalalabad	0.225	0.037037	1	27	MAS-192	15	
124		*Robinia pseudoacacia* L.	Kekar	Resin, wood, legumes	Paste	Jalalabad	0.35	0.076923	2	26	MAS-206	15	
125		*Astragalus zanskarensis* Bunge	Shukpa	Leaf, stem	Paste, ash	Skardu	0.2	0.25	2	8	MAS-386	39	
126		*Trifolium fragiferum* L.	Gul-e-Nasreen	Leaf, flower	Direct	Skardu	0.233333	0.142857	2	14	MAS-413	38	
127		*Trifolium pratense* L.	Chita-batta, Ol, Jangli shaftal	Flower	Powder	Astore, Hunza, Kargha, Skardu	0.2125	0.078571	6	76	MAS-017, MAS-147, MAS-260, MAS-429	9, 13, 14, 40, 41	
128		*Cicer microphyllum* Benth.	Stranjungstwa	Whole plant		Astore, Kargha, Skardu	0.230556	0.087446	3	35	MAS-025, MAS-267, MAS-437	13	
129		*Medicago sativa* L.	Ucharg, Ishfit	Whole plant	Direct, powder, decoction	Ghizer, Gojal	0.2	0.134921	9	70	MAS-058, MAS-115	16, 17, 37	
130		*Sophora mollis* (Royle) Baker	Khakhul, Popshing, Pushool	Leaf (K, Gh), leaf, seed (S), whole plant (H)	paste, powder, decoction (S)	Ghizer, Gojal, Hunza, Kargha, Skardu	0.243333	0.10857	10	108	MAS-119, MAS-151, MAS-277, MAS-455	9, 12,16, 17, 38, 39	
131		*Glycyrrhiza glabra* L*.*	Shalako	Root, rhizome (Gh), rhizome (K)	Decoction, paste	Ghizer, Kargha	0.3	0.091954	12	145	MAS-079, MAS-285	11, 16, 37	
132		*Trigonella foenum-graecum* L.	Shamilik	Leaf (K), whole plant (S, Gh)	Direct, decoction	Ghizer, Kargha, Skardu	0.277778	0.111683	6	62	MAS-086, MAS-292, MAS-474	37, 38, 39	
133		*Astragalus psilocentros* Fisch.	BiowaCharchu, Biacharchoo, Sokhrus, Hapoocho	Leaf, stem (K, H), leaf, root, thorny branches (S),	Decoction, infusion (S)	Ghizer, Hunza, Kargha, Skardu	0.258333	0.153501	9	63	MAS-088, MAS-166, MAS-294, MAS-476	9, 12, 13, 38	
134	Linaceae	*Linum usitatissimum* L.	Human	Seed	Powder	Hunza	0.175	0.07874	10	127	MAS-134	9, 32, 33	
135	Lythraceae	*Punica granatum* L.	Danooh, Sio, Dolum, Danu	Fruit, root (K, S), flower, fruit, seed, bark (H)	Decoction, paste, powder, direct	Gojal, Hunza, Jalalabad, Skardu	0.26875	0.112524	19	220	MAS-125, MAS-169, MAS-227, MAS-479	9, 15, 17, 33, 38	
136	Malvaceae	*Malva neglecta* Wallr.	Shanishah	Whole plant	Powder, decoction	Gojal	0.125	0.071429	1	14	MAS-095	17	
137		*Abelmoschus esculentus* (L.) Moench	Bhindi	Seed, fruit	Infusion	Hunza, Jalalabad, Nagar	0.308333	0.064052	3	57	MAS-174, MAS-233, MAS-362	9	
138		*Morus nigra* L.	Kini Marooch	Whole plant	Decoction, paste, direct	Hunza	0.225	0.1	3	30	MAS-135	33	
139		*Ficus carica* L.	Faag, Faak	Fruit, stem latex (N, H, K), fruit (Gh)	Poultice, direct, powder, paste	Ghizer, Hunza, Jalalabad, Nagar	0.275	0.096875	16	203	MAS-070, MAS-158, MAS-224, MAS-355	9, 15, 16, 33	
140		*Morus alba* L.	Marooch, Shae Marooch	Whole plant	Decoction, paste, direct	Hunza, Jalalabad	0.275	0.065763	7	119	MAS-171, MAS-230	9, 15, 32, 33	
141	Nitrariaceae	*Peganum harmala* L.	Spandur, Isman, Ispandure, Supandour	Whole plant (N, H), seed (Gh, S)	Powder, decoction, paste (H)	Ghizer, Gojal, Hunza, Nagar, Skardu	0.288333	0.097728	14	143	MAS-063, MAS-120, MAS-352, MAS-456	9, 16, 17, 33, 38, 53	
142	Nyctaginaceae	*Mirabilis jalapa* L.	Gul-e-Abbas	Flower	Paste	Hunza, Kargha	0.1875	0.142857	2	14	MAS-176, MAS-302	40, 41	
143	Oleacea	*Fraxinus hookeri* Wenz.	Kasunar	Bark, wood	Decoction	Jalalabad	0.275	0.047619	2	42	MAS-195	15	
144		*Fraxinus xanthoxyloides* (G.Don) Wall. ex A.DC.	Kasunar	Bark, wood	Decoction	Jalalabad	0.175	0.055556	2	36	MAS-196	15	
145		*Olea ferruginea* Wall. ex Aitch.	Kawoo	Leaf, wood, bark	Direct, decoction	Jalalabad	0.2	0.071429	1	14	MAS-200	15	
146	Onagraceae	*Epilobium latifolium* L.	Pondol	Leaf, flower	Paste, decoction	Skardu	0.233333	0.071429	1	14	MAS-390	12	
147	Orchidaceae	*Dactylorhiza hatagirea* (D.Don) Soó	Narmada	Root, rhizome	Powder	Gojal	0.15	0.090909	1	11	MAS-094	17	
148	Orobanchaceae	*Pedicularis cheilanthifolia* Schrenk	Serfo spanthing	Leaf	Decoction	Skardu	0.3	0.166667	2	12	MAS-396	12	
149		*Pedicularis pectinatiformis* Bonati	Sunpo spanthing	Leaf	Infusion	Skardu	0.233333	0.157895	3	19	MAS-397	12	
150	Papaveraceae	*Corydalis crassifolia* Royle	Sackros/Zarvosh	Whole plant		Gojal	0.2	0.142857	1	7	MAS-093	17	
151		*Papaver somniferum* L.	Mardakhaw	Latex	Decoction	Hunza	0.175	0.115385	3	26	MAS-136	9, 33	
152	Pinaceae	*Pinus roxburghii* Sarg.	Chirpine	Resin, wood	Paste, powder, direct	Ghizer	0.233333	0.121212	4	33	MAS-047	37	
153		*Picea smithiana* (Wall.) Boiss.	Kachul	Resin, wood	Powder, decoction	Jalalabad	0.2	0.071429	1	14	MAS-201	15	
154		*Pinus gerardiana* Wall.ex Lamb.	Cheenh	Resin, wood, leaf	Decoction, direct, paste, powder (k)	Jalalabad	0.175	0.096774	3	31	MAS-202	15	
155		*Pinus wallichiana* A.B.Jacks.	Cheenh	Resin, wood, leaf	Infusion, powder	Nagar	0.175	0.142857	3	21	MAS-340	15	
156	Plantaginaceae	*Plantago ovata* Forssk.	Ispaghol	Seed, leaf, root	Powder, infusion	Ghizer	0.233333	0.069767	3	43	MAS-048	16	
157		*Picrorhiza kurroa* Royle ex Benth.	Karroo	Leaf, bark, root, rhizome	Paste	Kargha	0.2	0.130435	3	23	MAS-244	11	
158		*Plantago major* L*.*	Shiltive, Boqna	Root, seed, leaf (K), seed (S), leaf, seed (Gh, H)	Direct, decoction, oil (S)	Ghizer, Hunza, Kargha, Skardu	0.2875	0.104482	9	127	MAS-073, MAS-161, MAS-282, MAS-466	9, 14, 33, 37, 38, 53	
159		*Plantago lanceolata* L.	Sman Hrswa, Sepgilk, Yeeps	Flower, leaf (S), leaf, seed (H)	Decoction, infusion, paste (S), ash (H)	Gojal, Skardu	0.295833	0.121667	6	49	MAS-131, MAS-485	12, 17	
160	Poaceae	*Zea mays* L.	Makayee	Fruit	Direct	Hunza	0.125	0.142857	1	7	MAS-139	9, 33	
161		*Cymbopogon jwarancusa* (Jones) Schult.	Izkhar Makki	Flower	Decoction	Kargha	0.175	0.133333	2	15	MAS-242	40, 41	
162		*Pennisetum glaucum* (L.) R.Br.	Cha soq	Stem	Direct	Kargha	0.175	0.0625	1	16	MAS-243	39	
163		*Saccharum bengalense* Retz.	Phoroo	Root, stem	Powder	Kargha	0.175	0.190476	4	21	MAS-247	14	
164		*Hordeum vulgare* L.	Cha Fay, York	Seed	Powder	Gojal, Kargha, Skardu	0.263889	0.161905	4	24	MAS-129, MAS-299, MAS-483	17, 39	
165		*Avena sativa* L.	Nas Choo, Sheshar	Seed (S), seed, leaf (H)	Decoction	Hunza, Skardu	0.270833	0.144796	5	47	MAS-190, MAS-496	9, 39	
166	Polygonaceae	*Rheum tibeticum* Maxim. ex Hook. f.	Sheepod	Stem	Direct	Gojal	0.2	0.142857	1	7	MAS-102	17	
167		*Bistorta amplexicaulis* (D.Don) Greene	Onbu	Root	Powder, decoction, infusion	Skardu	0.266667	0.214286	3	14	MAS-387	12	
168		*Polygonum affine* D. Don.	Strin mindoq	Root, flower	Decoction, infusion	Skardu	0.4	0.142857	3	21	MAS-398	12	
169		*Polygonum tataricum* L.	Bro Kho-Bro	Leaf, seed	Powder, decoction	Skardu	0.233333	0.157895	3	19	MAS-399	38	
170		*Rheum spiciforme* Royle	Khakhol	Leaf, root	Direct, powder	Skardu	0.266667	0.214286	3	14	MAS-405	39	
171		*Rumex chalepensis* Mill.	Sa-shing	Root	Decoction	Skardu	0.266667	0.083333	1	12	MAS-408	38	
172		*Fagopyrum esculentum* Moench	Bro, Ghiawas, Stabro, Baraw	Seed (K, H, A), leaf, seed (S)	Direct, paste, powder	Astore, Hunza, Kargha, Skardu	0.195833	0.150985	12	89	MAS-016, MAS-146, MAS-259, MAS-428	9, 13, 33, 38	
173		*Rumex nepalensis* Spreng.	Churkeen, Rashona	Root (K), leaf (A)	Paste	Astore, Kargha	0.229167	0.142857	2	14	MAS-021, MAS-263, MAS-433	13, 14	
174		*Rheum australe* D. Don	Shoot, Lachu	Root (K, A), leaf, root, stem (S)	Powder, infusion, decoction	Astore, Kargha, Skardu	0.280556	0.155556	8	50	MAS-030, MAS-272, MAS-442	12, 13	
175		*Oxyria digyna* (L.) Hill	Span Harswa, Skyurbutaq	Leaf (S), aerial (A)	Powder, decoction	Astore, Skardu	0.233333	0.177778	4	24	MAS-036, MAS-448	12, 38	
176		*Rheum emodi*	Jarochuntal, Chontal	Whole plant	Decoction	Ghizer, Hunza	0.258333	0.120909	5	61	MAS-068, MAS-156, MAS-461	9, 16	
177		*Rumex hastatus* D. Don	Churki	Whole plant (K), leaf, root, stem, fruit (Gh)	Direct, decoction, powder (Gh)	Ghizer, Kargha	0.3	0.093168	3	53	MAS-080, MAS-286	14, 16, 37	
178		*Bistorta affinis* (D.Don) Greene	Buma	Leaf	Powder	Kargha, Skardu	0.204167	0.142857	2	14	MAS-322, MAS-507	39	
179		*Polygonum hydropiper* L.	Thangmarcy	Leaf (K), aerial (S)	Decoction	Kargha, Skardu	0.2625	0.188235	5	27	MAS-333, MAS-518	14, 38	
180	Primulaceae	*Primula macrophylla* D. Don	Benufsha	Whole plant	Decoction, powder	Gojal	0.175	0.083333	1	12	MAS-101	17	
181		*Primula denticulata* Sm.	Daoo	Leaf, root	Decoction, powder, infusion (s)	Skardu	0.4	0.2	4	20	MAS-401	12	
182		*Primula farinose* L.	Spangpunar	Flower	Paste, decoction	Skardu	0.366667	0.142857	2	14	MAS-402	38	
183	Rananculaceae	*Clematis baltistanica* Qureshi & Chaudhri	Margush, Murgushi, Chindrik	Leaf, flower (H), whole plant (K)	Paste	Gojal, Jalalabad, Kargha	0.208333	0.086652	6	76	MAS-127, MAS-228, MAS-298	9, 14	
184		*Aconitum nepellus* L.	Booma, Sai booma	Flower, leaf (N, H, K), whole plant (K), aerial (S)	Direct	Kargha, Nagar, Skardu	0.2	0.231481	7	31	MAS-316, MAS-378, MAS-501	11, 38, 53	
185		*Ranunculus trichophyllus* Chaix ex Vill*.*	Threadleaf crowfoot	Whole plant	Paste, infusion	Kargha	0.175	0.111111	2	18	MAS-246	14	
186		*Thalictrum foetidum* L.	Momeran	Leaf	Direct	Kargha	0.225	0.142857	1	7	MAS-248	39	
187		*Aconitum violaceum* Jacquem. Ex. Stapf	Booma	Root	Decoction, powder	Skardu	0.233333	0.25	4	16	MAS-379	12	
188		*Aquilegia fragrans* Benth.	Karfo Koo-kuk	Leaf, flower	Paste, decoction	Skardu	0.233333	0.133333	2	15	MAS-382	38	
189		*Aquilegia pubiflora* Wall. Ex Royle	Koo-kuk	Leaf, flower	Paste	Skardu	0.233333	0.111111	2	18	MAS-383	38, 53	
190		*Thalictrum foliolosum* DC.	Momyrun	Root	Decoction	Skardu	0.4	0.142857	2	14	MAS-412	38	
191		*Delphinium brunonianum* Royle	Makhoting	Leaf, flower (K), whole plant (S, A)	Decoction, powder (S), infusion (S)	Astore, Kargha, Skardu	0.269444	0.132937	8	75	MAS-026, MAS-268, MAS-438	12, 13, 38, 39	
192		*Pulsatilla wallichiana* (Royle) Ulbr.	Zgiongmonana Loqparimandoq	Flower	Powder	Astore, Skardu	0.316667	0.142857	2	14	MAS-038, MAS-450	13	
193	Rosaceae	*Comarum salesovianum* (Stephan) Asch. & Graebn.	Noghurdoom woosh	Flower		Gojal	0.175	0.142857	1	7	MAS-092	9	
194		*Potentilla eriocarpa* Wall. ex Lehm.	Amber	Leaf, flower		Gojal	0.1	0.142857	1	7	MAS-099	17	
195		*Potentilla microphylla* D. Don	Zatspirg	Leaf, seed		Gojal	0.175	0.222222	2	9	MAS-100	17	
196		*Rubus irritans* Focke	Icheejeh	Fruit	Direct	Jalalabad	0.325	0.061224	3	49	MAS-207	15	
197		*Potentilla argyrophylla* Wall. ex Lehm.	Serfo Harswa	Whole plant	Paste	Skardu	0.466667	0.142857	1	7	MAS-400	12	
198		*Rosa webbiana* Wall. ex Royle	Shighaye, Sia marpho, Chereer, Sia sarfo	Bark, wood (K), flower, bark (S), fruit, seed, wood (H, A)	Decoction	Astore, Gojal, Hunza, Jalalabad, Skardu	0.238333	0.103989	10	116	MAS-010, MAS-110, MAS-144, MAS-422	9, 13, 15, 17, 38, 53	
199		*Prunus armeniaca* L.	Jui, Jaroty, Chooli	Fruit, kernel, oil	Direct, oil, powder, paste	Astore, Hunza, Jalalabad, Kargha, Nagar, Skardu	0.276389	0.082586	49	706	MAS-015, MAS-145, MAS-218, MAS-427	9, 13, 15, 32, 33, 38, 39	
200		*Spiraea canescens* D.Don	Darah, Skhsi	Flower, stem, wood	Oil, decoction	Astore, Jalalabad	0.175	0.066667	3	52	MAS-018, MAS-219	13, 15	
201		*Potentilla salesoviana* Steph.	Sniarmastwa, Karfo mindoq	Flower	Infusion (s), paste	Astore, Kargha, Skardu	0.222222	0.116883	8	78	MAS-029, MAS-271, MAS-441	12, 13,38	
202		*Rosa brunonii* Lindl.	SiaMarpho, Siya	Bark	Decoction, powder (k), infusion (s)	Astore, Kargha, Skardu	0.216667	0.142857	3	21	MAS-031, MAS-273, MAS-443	12, 13	
203		*Prunus dulcis* (Mill.) D.A.Webb	Badum, Balth, Kono, Stargi mar	Kernel, flower	Direct, oil, paste, decoction (s)	Ghizer, Hunza, Jalalabad, Kargha, Nagar, Skardu	0.231944	0.11893	21	194	MAS-069, MAS-223, MAS-354, MAS-462	15, 16, 32, 39	
204		*Rosa indica* L.	Ghulab	Flower	Paste, oil	Ghizer, Hunza, Skardu	0.313889	0.090149	6	62	MAS-076, MAS-164, MAS-469	32, 37, 38	
205		*Malus domestica* Borkh.	Skamkooshu	Fruit	Powder	Kargha, Skardu	0.270833	0.142857	2	14	MAS-332, MAS-517	39	
206		*Potentilla bifurca* L.	Tarqan	Flower (K), aerial (S)	Infusion (S), decoction (K)	Kargha, Skardu	0.345833	0.103343	4	54	MAS-334, MAS-519	12, 38, 39	
207		*Prunus persica* (L.) Batsch	Takushu Choo	Fruit	Paste (k), decoction (s)	Kargha, Skardu	0.2375	0.171429	3	17	MAS-335, MAS-520	39, 53	
208	Salicaceae	*Salix babylonica* L.	Muchoor	Leaf, bark, seed, gum	Decoction, paste, direct	Hunza	0.125	0.142857	4	28	MAS-138	9	
209		*Salix acmophylla* Boiss.	Brawoon	Leaf, bark, stem, branches	Decoction, paste	Jalalabad	0.275	0.074627	5	67	MAS-208	15, 53	
210		*Salix denticulata* Andersson	Brawoon	Leaf, bark, stem, branches	Decoction, paste, direct	Jalalabad	0.225	0.083333	5	60	MAS-209	15	
211		*Salix iliensis* Regel	Brawoon	Leaf, bark, stem, branches	Decoction, paste, direct	Jalalabad	0.175	0.09434	5	53	MAS-210	15	
212		*Salix sericocarpa* Andersson	Brawoon	Leaf, bark, stem, branches	Decoction, paste, direct	Jalalabad	0.175	0.096154	5	52	MAS-211	15	
213		*Salix turanica* Nasarov	Brawoon	Leaf, bark, stem, branches	Decoction, paste, direct	Jalalabad	0.2	0.1	5	50	MAS-212	15	
214		*Salix alba* L.	Mori Bayao, Bayo	Leaf, bark	Decoction, paste, direct	Ghizer, Kargha	0.225	0.099462	5	55	MAS-081, MAS-287	14, 16	
215		*Populus alba* L.	Fulsoo, Turaq	Leaf, wood (K), leaf (H)	Decoction	Hunza, Jalalabad	0.1375	0.107143	2	21	MAS-172, MAS-231	9, 15, 33	
216		*Populus nigra* L.	Jerpa	Leaf	Decoction	Hunza, Jalalabad, Nagar	0.166667	0.119048	3	28	MAS-175, MAS-234, MAS-363	15, 33	
217		*Salix tetrasperma* Roxb.	Byao, Bew	Leaf, bark	Decoction, paste, direct	Hunza, Kargha	0.2	0.107143	2	21	MAS-177, MAS-303	9, 14	
218	Saxifragaceae	*Saxifraga hirculus* L.	Sitbark	Whole plant	Decoction	Gojal	0.15	0.117647	4	34	MAS-103	17	
219		*Bergenia himalacia* Boriss.	Sanspur	Root	Powder, decoction	Kargha	0.175	0.133333	2	15	MAS-241	9, 11, 12, 16, 33, 53	
220		*Bergenia stracheyi* (Hook.f. & Thomson) Engl.	Sasper, Khichlay	Root, leaf	Infusion, powder, paste, direct	Ghizer, Hunza, Nagar, Skardu	0.25625	0.14944	18	124	MAS-075, MAS-163, MAS-357, MAS-468	9, 11, 12, 16, 33	
221		*Bergenia ciliata* (Haw.) Sternb.	Shafus, Shaphus	Leaf (K), leaf, seed (S)	Powder, decoction, direct	Kargha, Skardu	0.308333	0.162338	12	75	MAS-321, MAS-506	12, 13, 39	
222	Solanaceae	*Solanum nigrum* L.	Gabeeli, Gabilo, Drumbashokhlo	Whole plant (N,Gh), leaf, fruit (K), fruit, seed (H), fruit (A)	Direct, decoction (K), powder (Gh)	Astore, Ghizer, Gojal, Kargha, Nagar	0.243333	0.125426	20	190	MAS-005, MAS-053, MAS-252, MAS-343	13, 14, 16, 17, 37	
223		*Hyoscyamus niger* L.	Landungstwa	Seed	Paste, poultice, decoction	Astore, Kargha, Skardu	0.25	0.15873	3	19	MAS-027, MAS-269, MAS-439	13, 53	
224		*Datura stramonium* L.	Daturo, Datura	Seed (H), flower, fruit, seed, leaf (Gh, K)	Decoction, ash (K), paste (K)	Ghizer, Hunza, Kargha	0.266667	0.105286	13	138	MAS-071, MAS-159, MAS-280	9, 14, 16, 33, 37, 53	
225		*Capsicum annuum* L.	Marooch	Fruit	Direct	Hunza, Nagar	0.2375	0.065613	5	101	MAS-187, MAS-370	9	
226	Tamaricaceae	*Myricaria squamosa* Desv.	Targ	Leaf, flower	Powder	Gojal	0.175	0.178571	5	28	MAS-097	17	
227	Thymelaeaceae	*Daphne mucronata* Royle	Nirko	Leaf, fruit, wood	Paste, poultice	Jalalabad	0.275	0.088889	4	45	MAS-193	15	
228	Urticaceae	*Urtica dioica* L.	Khaeshing	Whole plant (S, K), leaf, root (Gh)	Direct, decoction, paste (S)	Ghizer, Kargha, Skardu	0.236111	0.132675	12	149	MAS-087, MAS-293, MAS-475	13, 37, 53	
229	Violaceae	*Viola serpens* Wall. ex Ging.	Skora mindoq, Lillo	Flower (S), whole plant (Gh, H)	Decoction	Ghizer, Hunza, Skardu	0.258333	0.124008	6	58	MAS-077, MAS-165, MAS-470	9, 16, 33, 38	
230	Zingiberacaceae	*Curcuma longa* L.	Halichi	Stem	Powder	Hunza, Nagar	0.275	0.088933	4	45	MAS-188, MAS-371	32	
231	Zygophyllaceae	*Tribulus terrestris* L.	Kokoloq, Kokoring, Huk ga kurice	Whole plant (Gh, K, A), seed (S), fruit (H)	Paste, decoction	Astore, Ghizer, Hunza, Kargha, Skardu	0.23	0.142857	9	63	MAS-008, MAS-056, MAS-255, MAS-420	9, 13, 33, 37, 38, 39	

### Medicinal systems and affiliations

This region has already witnessed invasion by different cultures and practices [3, 46–51]. The passes created by the Indus River system in Hunza, Shigar, Shyjok, Ghizer, Gilgit, and Astore valleys served as the main travel routes for such invasions and exchanges as Gilgit got its famous name “gate to India” [3]. This region witnessed influence from Chinese, Tibetan, ancient Indian, and Unani systems [3, 46]. This influence was reflected during the field survey while recording the uses and modes of use of medicinal plants.

Fifty percent of the participants were able to answer the question related to influence of medicinal systems on the indigenous knowledge existing in the region; where 28% referred to Chinese influence, 23% chose Indian subcontinent while 18 and 14% selected Scythian/Transoxianan, and ancient Greek influence on the traditional medicinal system. It is worth mentioning that 41% of these participants referred to a mix of at least two of these systems in the current traditional medicinal system. Most of the participants from Astore and Skardu mentioned Indian subcontinent followed by Scythian/Transoxianan and ancient Greek influence while participants from Hunza, Nagar, Gilgit, and Ghizer ranked Chinese influence on top followed by Indian subcontinent, Scythian/Transoxianan, and ancient Greek. The market players on the other hand opted for a mix of all these systems together as they deal with customers from the whole region and are exposed to all medicinal systems prevailing in the region. Another reason for a mixed system described by the market players is the fact that these markets were traditionally placed at regional centers, thus were exposed to THPs representing different systems. Their experiences with these THPs and fulfilling their demands made them acquire traits from all the systems. Although a clear boundary could not be drawn between these systems, it is likely that medicinal practices in Hunza, Nagar, Ghizer, and Gilgit were influenced by traditional Chinese medicine (TCM) system while the remaining part of study area was dominated by a mix of Ayurveda and Unani systems.

### Discriminant analysis (DA)

**Fig. 8:**
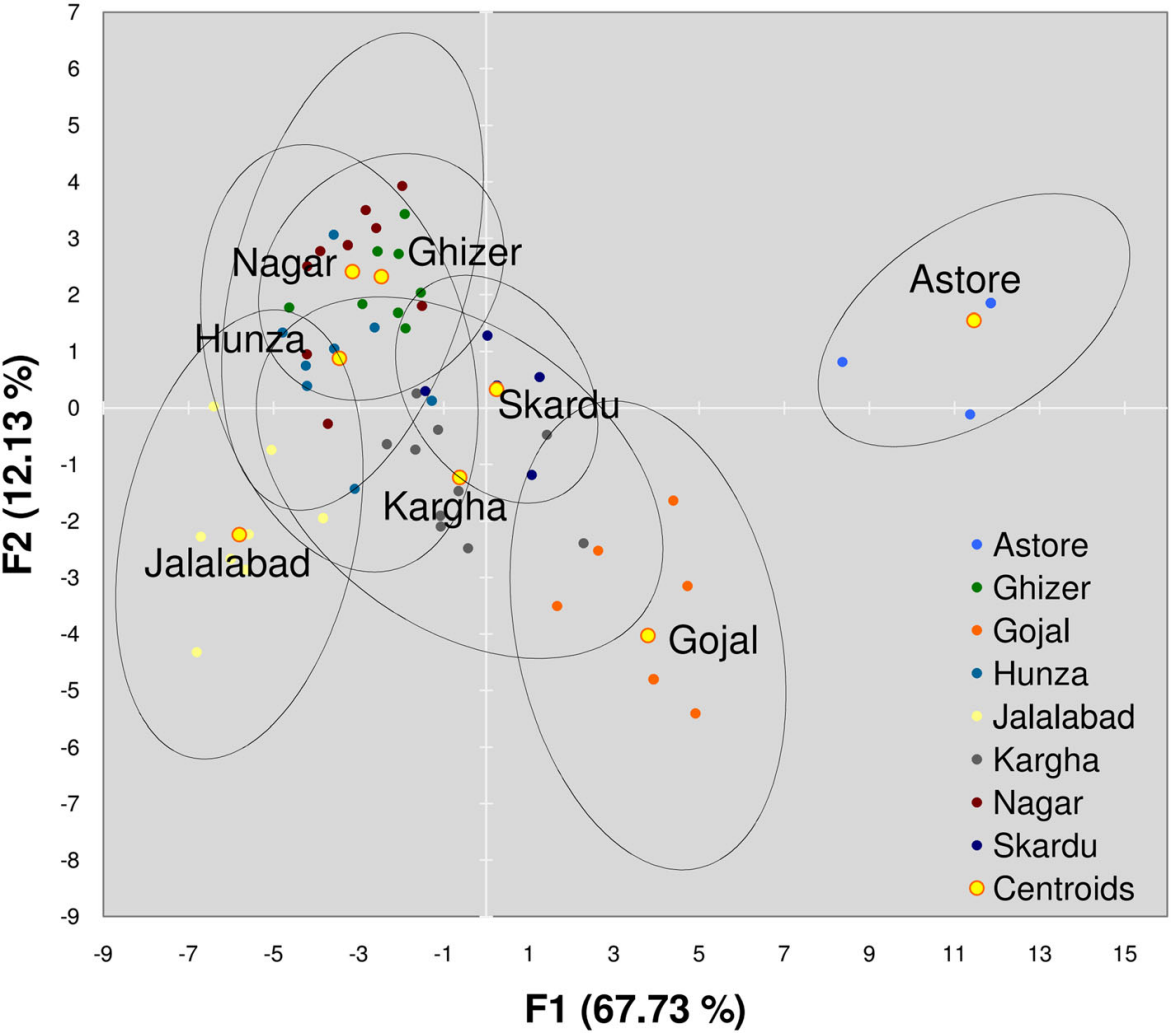
Percentage of the parts of plants used for medicinal purposes

Discriminant analysis revealed that Astore was distinct from other surveyed areas whereas some overlap can be observed in other surveyed areas (Fig. 8). Gojal showed similarity with Skardu and Kargha, while Jalalabad showed few similarities with Kargha and Hunza. Hunza, Nagar, and Ghizer showed very similar traits while sharing few similarities with Skardu, Kargha, and Jalalabad. This analysis mainly considered top 10 medicinal plants used and the parameters calculated from each location. This does not represent linkage with healing systems but separates geographical location, tribal representation, and connectivity to other parts of the region, e.g., China, India, and routes to Western Asia. It also reflects on migration and integration of the local tribes as well as those coming from outside and provides an insight on the influence of different invaders, travelers, and businessmen.

Plant species have responded to latitudinal and elevational changes in their habitat and adjusted over time, yet the increasingly rapid pace of these changes is challenging their adaptability and ability to respond [96–98]. The association of traditional knowledge with these species, trade potentials, and transfer of knowledge from old to new generation will have a direct effect on the conservation of plant species and associated TK [90, 99–103].

### Key discussion points

The environment and climatic conditions of Gilgit-Baltistan make it geographically one of the best locations for growth and nourishment of medicinal plants [6, 9, 34, 104]. Traditional medicinal practices hold a significant place in the lives of the local communities. The markets at Gilgit and Skardu are serving as trade centers for medicinal plants from the whole region, and the wholesalers stationed here are responsible for small-scale trade in important plant species in both local and national markets. The study shows that most of the large herbal medicine production companies in Pakistan rely on supply of medicinal plants from Indian territory—representing the same region across the border—indicating that the production and trade of medicinal plants is well organized across the border. The trade of medicinal plants in Pakistan is informal, with little to none state interventions and incentives [35, 105]. The high marketability of medicinal plants has led many local people to over-exploit this valuable resource. Over-exploitation of medicinal plant species is widespread across the region, exacerbated by some local people attempting to maximize financial benefits in a single harvest, with little concern for the ramifications for subsequent years [9, 30, 34, 42].

This study clearly reveals the importance and contributions of the THPs and retailers as well as the transfer of knowledge within the families from elders to the younger generation for the retention of indigenous medicinal knowledge, where and when to acquire a particular species and the utilization of medicinal plants. The THPs preserved existing knowledge and showed a great deal of openness to knowledge sharing. They attributed the loss of knowledge between generations, not to any failure of their own to impart knowledge, but rather to a lack of learning aptitude in the younger generation [34, 106]. Retailers have adapted well to the demands of different ethnic and tribal groups. These factors point towards a high level of cooperation, collaboration, and openness to knowledge exchange amongst the ethnicities and tribes of Gilgit-Baltistan. Local public and private institutes can therefore play a vital role in clustering the knowledge and bridging the gaps by providing platforms for recording, sharing, and disseminating traditional knowledge.

We found that Gilgit-Baltistan’s position as a gateway between the Central and South Asia caused its exposure to a number of traditional medicinal systems including the Ayurveda, traditional Chinese medicine, Unani, and Tibetan—which highly influenced traditional medicine knowledge in this region [3, 46]. Our study design and timeline restricted us from further exploration of these historical details. Therefore, we were not able to explore the timeline and actual contributions of these systems to local knowledge. However, it appears likely that medicinal practices in Hunza, Nagar, Ghizer, and Gilgit were influenced by traditional Chinese medicine (TCM) system while the remaining part of the study area was dominated by a mix of Ayurveda and Unani systems. This is an interesting finding and deserves further research. Most parts of Pakistan are primarily relying on a mix of Unani and Ayurveda medicinal systems—a combination which is rarely found elsewhere [107]. A dedicated study exploring the approaches followed by these medicinal systems, their complementarities, and differences could lead to the generation of highly valuable scientific findings that could contribute to the communities relying on these systems globally.

With the involvement of multiple stakeholders (the relevant local government departments, herbal medicine-producing companies, THPs, and the interest of the national government), medicinal plants and associated traditional knowledge from Gilgit-Baltistan can make a substantial contribution to traditional health practices at a national level as well as contribute significantly to the national market and the livelihood resources of local communities. Proper licensing will allow the THPs to legally practice, document, and disseminate their knowledge. The concerned government departments can provide a platform for THPs from the region to get registered and licensed as hakims [39]. Our effort to involve school students in the collection of data is a way of exposing the younger generation to identification of their resources and developing their interest in traditional knowledge and why it is important to ensure its transfer to them from the older generation. Such a consortium will also prove beneficial for the production of medicinal plants on a commercial scale, their sustainable utilization, and organizing refreshers on different aspects associated with medicinal plant resources for the local THPs, retailers, and collectors in order to ensure an optimal and efficient utilization of the available resources.

These points are of utmost importance when it comes to the conservation and transfer of traditional medicinal knowledge to future generations. Worldwide, patients are increasingly opting for medications involving traditional techniques, herbal medicine, and meditation [108, 109]. Gilgit-Baltistan has natural medicinal resources, a vast indigenous knowledge bank, and most importantly one of the best mountainous landscapes for tourism and meditation. It is doubtless the best option to be considered for developing into a sanctuary through government interventions. Gilgit-Baltistan, considered to be home to the ideology of “SHANGRI LA” with its abundant natural resources, can provide a home to those who seek medication through centuries-old traditional knowledge, sacredly transferred from one generation to another.

## Conclusions and recommendations

The diverse plant resources and the geographical importance of the region for trade and travel routes, historically made Gilgit-Baltistan a hotspot for cultural, religious, and traditional knowledge exchange. Being part of an ancient trade route, the resident communities adapted and upgraded their traditional healing systems through interactions with the Indian subcontinent, China, Scythia, Transoxiana, and Ancient Greece. This influence and amalgamation of Chinese, Ayurveda, Unani, and Tibetan medicinal systems is apparent in local traditional knowledge. Our study revealed that most of the local people still rely on indigenous healing practices. Higher knowledge and use of medicinal plants is retained in the areas that also serve as main trade centers in the region. The trade of medicinal plants in the region is the one key factor in retaining traditional knowledge on medicinal plant utilization. This continued reliance on medicinal plants shows the significance of these traditional practices. A thorough evaluation is needed by ethno-pharmacologists and other concerned institutions working for public health and hygiene, especially focusing on THPs, market actors and old folk from the region. For strategies to be devised for market exploration, raising awareness, and continuity of TK, involvement is required from Government institutions, research organizations, NGOs, donors, and the private sector.

## Additional files

Additional file 1: Primary data on medicinal plants and their uses collected during the field survey. (XLSX 636 kb)

Additional file 2: RFC and UV of species reported from Central Hunza. (XLSX 14 kb)

Additional file 3: RFC and UV of species reported from Ghizer. (XLSX 14 kb)

Additional file 4: RFC and UV of species reported from Gojal Hunza. (XLSX 13 kb)

Additional file 5: RFC and UV of species reported from Jalalabad. (XLSX 13 kb)

Additional file 6: RFC and UV of species reported from Kargha. (XLSX 17 kb)

Additional file 7: RFC and UV of species reported from Nagar. (XLSX 13 kb)

Additional file 8: RFC and UV of species reported from Skardu. (XLSX 19 kb)

Additional file 9: RFC and UV of species reported from Astore. (XLSX 13 kb)

Additional file 10: Top 10 species from each location for number of uses, ailment categories, RFC, and UV. (XLSX 14 kb)

Additional file 11: List of publications from the region reporting on medicinal plants and their uses. (XLSX 12 kb)

Additional file 12: Ailment categories. (XLSX 14 kb)

## Abbreviations

CPEC: China-Pakistan Economic Corridor
DA: Discriminant analysis
FC: Frequency of citation
FGD: Focused group discussion
HH: Household
HKH: Himalaya Karakoram Hindukush Mountain Range
ICF: Informant consensus factor
KKH: Karakoram Highway
MAPs: Medicinal and aromatic plants
NGO: Non-Governmental Organization
RFC: Relative frequency of citation
TCM: Traditional Chinese medicine
THP: Traditional health practitioner
TK: Traditional knowledge
UV: Use value
